# Macroscale patterns of oceanic zooplankton composition and size structure

**DOI:** 10.1038/s41598-021-94615-5

**Published:** 2021-08-03

**Authors:** Manoela C. Brandão, Fabio Benedetti, Séverine Martini, Yawouvi Dodji Soviadan, Jean-Olivier Irisson, Jean-Baptiste Romagnan, Amanda Elineau, Corinne Desnos, Laëtitia Jalabert, Andrea S. Freire, Marc Picheral, Lionel Guidi, Gabriel Gorsky, Chris Bowler, Lee Karp-Boss, Nicolas Henry, Colomban de Vargas, Matthew B. Sullivan, Tara Oceans Consortium Coordinators, Silvia G. Acinas, Marcel Babin, Peer Bork, Emmanuel Boss, Chris Bowler, Guy Cochrane, Colomban de Vargas, Gabriel Gorsky, Lionel Guidi, Nigel Grimsley, Pascal Hingamp, Daniele Iudicone, Olivier Jaillon, Stefanie Kandels, Lee Karp-Boss, Eric Karsenti, Fabrice Not, Hiroyuki Ogata, Nicole Poulton, Stephane Pesant, Jeroen Raes, Christian Sardet, Sabrina Speich, Lars Stemmann, Matthew B. Sullivan, Shinichi Sunagawa, Patrick Wincker, Lars Stemmann, Fabien Lombard

**Affiliations:** 1 Sorbonne Université, CNRS, Laboratoire d’Océanographie de Villefranche, 06230 Villefranche-sur-mer, France; 2 Ifremer, Centre Bretagne, Unité Dynamiques des Ecosystèmes Côtiers, 29280 Plouzané, France; 3 ETH Zürich, Institute of Biogeochemistry and Pollutant Dynamics, 8092 Zürich, Switzerland; 4 Aix Marseille Univ., Université de Toulon, CNRS, IRD, MIO UM 110, 13288 Marseille, France; 5 Ifremer, Centre Atlantique, Unité Ecologie et Modèles Pour l’Halieutique, 44311 Nantes, France; 6 Departamento de Ecologia e Zoologia, Universidade Federal de Santa Catarina, Florianópolis, 88010970 Brazil; 7 Institut de Biologie de l’École Normale Supérieure (IBENS), CNRS, INSERM, PSL Université Paris, 75005 Paris, France; 8 Research Federation for the Study of Global Ocean Systems Ecology and Evolution, FR2022/Tara Oceans GOSEE, 75016 Paris, France; 9 School of Marine Sciences, University of Maine, Orono, 04469 USA; 10 Sorbonne Université, CNRS, Station Biologique de Roscoff, AD2M, UMR 7144, 29680 Roscoff, France; 11 Department of Microbiology and Civil, Environmental, and Geodetic Engineering, The Ohio State University, Columbus, 43214 USA; 12 Institut Universitaire de France, 75231 Paris, France; 13 Institute of Marine Sciences (ICM) – CSIC, Pg. Marítim de la Barceloneta, 37-49, 08003 Barcelona, Spain; 14 Takuvik Joint International Laboratory (UMI3376), Université Laval (Canada) – CNRS (France), Université Laval, Québec, QC G1V 0A6 Canada; 15 Structural and Computational Biology, European Molecular Biology Laboratory, Meyerhofstrasse 1, 69117 Heidelberg, Germany; 16 European Molecular Biology Laboratory, European Bioinformatics Institute (EMBL-EBI), Wellcome Trust Genome Campus, Hinxton, Cambridge, UK; 17 CNRS Biologie Intégrative Des Organismes Marins (BIOM), UMR7232, 1 avenue Pierre Fabre, 66650 Banyuls-sur-Mer, France; 18 Sorbonne Université, Observatoire Océanologique de Banyuls-Sur-Mer, 1 avenue Pierre Fabre, 66650 Banyuls-sur-Mer, France; 19 Stazione Zoologica Anton Dohrn, Villa Comunale, 80121 Naples, Italy; 20 Génomique Métabolique, Genoscope, Institut de Biologie François Jacob, Commissariat à l’Énergie Atomique (CEA), CNRS, Université Évry, Université Paris-Saclay, Évry, France; 21 Directors’ Research, European Molecular Biology Laboratory, Meyerhofstrasse 1, 69117 Heidelberg, Germany; 22 Institute for Chemical Research, Kyoto University, Gokasho, Uji, Kyoto, 611-001 Japan; 23 Bigelow Laboratory for Ocean Sciences, East Boothbay, ME 04544 USA; 24 PANGAEA, Data Publisher for Earth and Environmental Science, University of Bremen, Bremen, Germany; 25 MARUM, Center for Marine Environmental Sciences, University of Bremen, Bremen, Germany; 26 Department of Microbiology and Immunology, Rega Institute, KU Leuven, Herestraat 49, 3000 Leuven, Belgium; 27 Center for the Biology of Disease, VIB, Herestraat 49, 3000 Leuven, Belgium; 28 Department of Applied Biological Sciences, Vrije Universiteit Brussel, Pleinlaan 2, 1050 Brussels, Belgium; 29 Sorbonne Université, CNRS, UMR 7009 Biodev, Observatoire Océanologique, 06230 Villefranche-sur-mer, France; 30 Department of Geosciences, Laboratoire de Météorologie Dynamique (LMD), Ecole Normale Supérieure, 24 rue Lhomond, 75231 Paris Cedex 05, France; 31 Laboratoire de Physique des Océans, UBO-IUEM, Place Copernic, 29820 Plouzané, France; 32 Department of Biology, Institute of Microbiology and Swiss Institute of Bioinformatics, ETH Zürich, Vladimir-Prelog-Weg 4, 8093 Zürich, Switzerland

**Keywords:** Ecology, Ocean sciences

## Abstract

Ocean plankton comprise organisms from viruses to fish larvae that are fundamental to ecosystem functioning and the provision of marine services such as fisheries and CO_2_ sequestration. The latter services are partly governed by variations in plankton community composition and the expression of traits such as body size at community-level. While community assembly has been thoroughly studied for the smaller end of the plankton size spectrum, the larger end comprises ectotherms that are often studied at the species, or group-level, rather than as communities. The body size of marine ectotherms decreases with temperature, but controls on community-level traits remain elusive, hindering the predictability of marine services provision. Here, we leverage *Tara* Oceans datasets to determine how zooplankton community composition and size structure varies with latitude, temperature and productivity-related covariates in the global surface ocean. Zooplankton abundance and median size decreased towards warmer and less productive environments, as a result of changes in copepod composition. However, some clades displayed the opposite relationships, which may be ascribed to alternative feeding strategies. Given that climate models predict increasingly warmed and stratified oceans, our findings suggest that zooplankton communities will shift towards smaller organisms which might weaken their contribution to the biological carbon pump.

## Introduction

Body size has been defined as a “master trait” for plankton as it is a morphological characteristic shared by organisms across taxonomy and that characterizes the functions performed by organisms in ecosystems^1,2^. It has a paramount effect on growth, reproduction, feeding strategies and mortality^3^. One of the oldest manifestations of the biogeography of traits was proposed over 170 years ago, namely Bergmann’s rule, in which field observations showed that larger species tend to be found at higher, colder latitudes^4^.

In the oceans, size is critical in determining trophic links in planktonic ecosystems and is thus a critical factor in regulating the efficiency of the biological carbon pump^5^. Body size is sensitive to changes in temperature due to the thermal dependence of physiological processes^6^. The plankton is mainly composed of ectotherms which are organisms that do not generate sufficient metabolic heat to elevate their body temperature, so their metabolic processes depends on external temperature^7^. Consequently, ectotherms grow more slowly and reach maturity at a larger body size in colder environments, which has long puzzled biologists because classic theories of life-history evolution predict smaller adult sizes in environments delaying growth^8^. This pattern of body size variation, known as the temperature-size rule (TSR^9^), has been observed for a wide range of ectotherms, including single-celled and multicellular species, invertebrates and vertebrates^8,10^.

The processes underlying the inverse relationship between body size and temperature remain to be identified^8^. Despite temperature playing a major role in shaping latitudinal variations in organism size, these patterns may also rely on complex interactions between physical, chemical and biological factors. For instance, oxygen supply plays a central role in determining the magnitude of ectothermic temperature-size responses, but it is hard to disentangle the relative effects of oxygen and temperature from field data because these two variables are often strongly inter-related in the surface ocean^11,12^.

The major drivers of community-level plankton size structure (i.e. distribution of individual body size in a given community) must be identified to effectively perform the ecological predictions that are progressively requested in a context of climate change^13^. Global patterns of phytoplankton biomass, size and community composition have been extensively studied thanks to satellite sensors that can detect phytoplankton pigments from space. Satellite observations showed that larger phytoplankton dominate in upwelling regions and at high latitudes where seasonal mixing regimes elicit higher macronutrients availability^14,15^. In contrast, zooplankton size structure and composition remain challenging to study in situ and remain poorly constrained by observations. Body size variations of planktonic copepods have been derived from literature-based relationships and have been found to display latitudinal patterns driven by variations in temperature and primary production^16^. Previous studies showed that temperature, rather than food availability, is the dominant variable in explaining variations in copepod body size^17^. Body size can be altered experimentally in the laboratory^18,19^. However, how these species-specific-based and/or laboratory-based observations can be transferred to the size structure of natural communities remains unclear. Knowing how size structure and abundance scale with changing abiotic conditions at the community level is critical because these factors determine the production and the functioning of the entire ecosystem^20^.

Here, we use plankton samples homogeneously collected at a macroscale during the *Tara* Oceans expeditions (2009–2013) that were analyzed with the ZooScan imaging system^21^ to document how zooplankton composition (i.e., the abundance of different groups) and size structure at the community level varies with latitude, temperature, oxygen, macronutrient concentrations, phytoplankton biomass and other ecosystem properties. We develop multivariate regression models to identify the underlying drivers of the global gradients of abundance and size structure for more than 30 zooplankton clades.

## Results

### Latitudinal patterns of zooplankton abundance and composition

**Figure 1:**
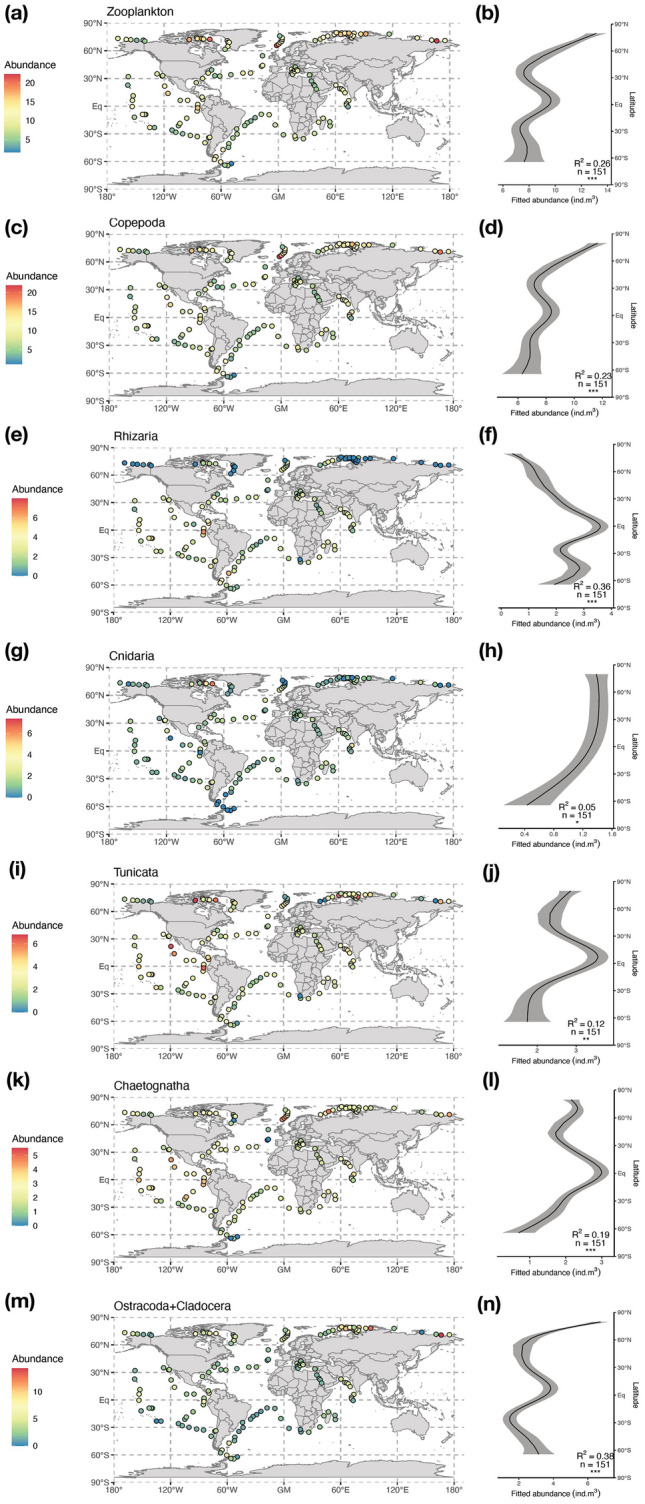
Maps and latitudinal patterns of the abundance (cubic-transformed ind m^3^) of (**a**,**b**) Total zooplankton, (**c**,**d**) Copepoda, (**e**,**f**) Rhizaria, (**g**,**h**) Cnidaria, (**i**,**j**) Tunicata, (**k**,**l**) Chaetognatha, and (**m**,**n**) Ostracoda + Cladocera observed in samples collected by the WP2 net. The solid curves on the right-hand side plots illustrate the prediction from the Generalized Additive Model (GAM) fitting abundance against latitude. The explanatory power of the GAM (adjusted R^2^), the number of samples used and the significance of the smooth term (p < 0.001 = ***, p < 0.01 = ***, p < 0.05 = *, p > 0.05 = ns) are reported on the plots. The grey ribbon illustrates the standard error of the GAM prediction.

Based on the Zooscan analysis of the WP2 (200 µm mesh), Bongo (300 µm mesh) and Régent (680 µm mesh) net samples, we found that most of the 36 zooplankton groups retained displayed significant latitudinal patterns of abundance (Fig. 1). Here, we focused on the significant patterns observed for total zooplankton and those broad groups displaying the highest contributions to total abundance (i.e., Copepoda, Rhizaria, Cnidaria, Tunicata, Chaetognatha and Ostracoda plus Cladocera) based on WP2 net samples, as this net showed the broadest spatial coverage (Supplementary Fig. S1). The spatial patterns were nonetheless consistent across all three nets and for all other groups (Supplementary Fig. S2). Total zooplankton and its main constituting groups displayed non-monotonic gradients of abundance with peaks in the Arctic and/or near the equator, and depressions in the tropical gyres. Zooplankton abundance was highest in the Arctic (Fig. 1a,b), north of 60°N, and decreased progressively towards the equator. A secondary peak was visible near the equator because of the relatively higher abundance in the eastern tropical Pacific Ocean. Zooplankton abundance decreased towards the Southern Ocean, whose few sampled stations displayed the lowest abundances.

Gradients in zooplankton abundance were clearly driven by copepods (Fig. 1c,d) as those dominated community composition (74% of total abundance in WP2 samples, 73% and 82% in the Bongo and Régent samples, respectively; Supplementary Doc. S3). Copepods displayed the same abundance pattern as total zooplankton but showed a slightly weaker tropical peak. The latter was actually more marked for other groups, especially the Rhizaria (Fig. 1e,f) that showed very low abundances towards the poles. Gelatinous groups displayed contrasted patterns. Latitudinal gradients were more marked for Tunicata (Fig. 1i,j) and Chaetognatha (Fig. 1k,l) than for total zooplankton as their abundance levels observed in tropical upwelling regions compete with those observed in the Arctic Ocean. Carnivorous jellyfishes (Cnidaria; Fig. 1g,h) displayed a weakly significant latitudinal pattern that was driven by higher abundances in the western Arctic Ocean. Eumalacostraca (i.e., macrozooplankton such as euphausiids, amphipods and decapods) also showed strong bimodal gradient but only in the Régent data, and pteropods showed no distinguishable latitudinal abundance pattern (Supplementary Fig. S2).

**Figure 2:**
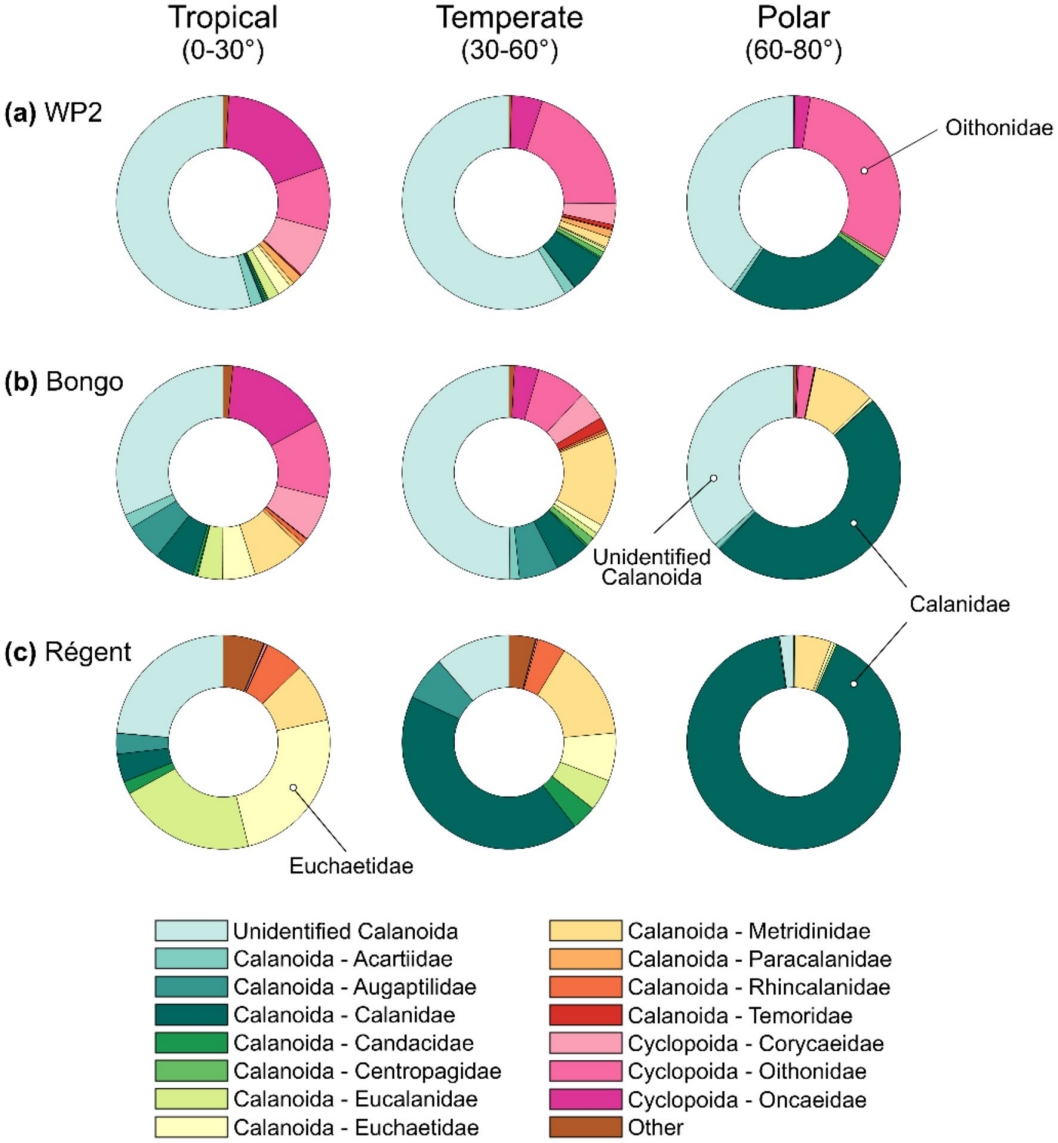
Variations in Copepoda community composition across the tropical (0–30°), temperate (30°–60°) and polar (> 60°) latitudinal bands, depicted through the changes in relative abundances of the copepod Orders (Calanoida, Cyclopoida and Poecilostomatoida) and Families sampled by the (**a**) Bongo net, (**b**) WP2 net, and (**c**) Régent net. Taxa with lower than 1% are not shown. Unidentified categories correspond to those organisms that could be assigned to an Order but not to a Family because of the limited resolution of the imaging system.

Considering the dominance of copepods in terms of abundances in the communities sampled, we examined the underlying latitudinal gradients in copepod order and family composition (Fig. 2). All nets (Fig. 2a–c) showed an increase in the relative contribution of calanoid families, and especially the large-bodied Calanidae, to the detriment of Cyclopoida (Oithonidae) and Poecilostomatoida (Oncaeidae, Corycaeidae and Sapphirinidae). The relative abundances of copepod families were more evenly distributed in the tropics than in the poles, reflecting gradients of decreasing copepod diversity with latitude (already documented by Ibarbalz et al.^22^). The variations in copepod abundance were driven by the increase in calanoids (mainly Calanidae) and oithonids towards the Arctic Ocean (Supplementary Fig. S2). Conversely, the following families showed clear abundance peaks in tropical regions (gyres or upwelling): Augaptilidae, Candaciidae, Corycaeidae, Eucalanidae, Euchaetidae, Oncaeidae, Paracalanidae, Sapphirinidae and Temoridae. The WP2 data showed less marked variations (Fig. 2a) as this net better samples the smaller Poecilostomatoida and Cyclopoida. The Régent net captured a lower quantity of unidentified Calanoida (Fig. 2c) as the WP2 and Bongo nets (Fig. 2b) as the relatively coarse mesh of this net is not able to retain smaller organisms, a pattern that was found across all zooplankton groups (Supplementary Doc. S4). The Bongo net showed lower zooplankton abundances than the WP2 net because of its coarser mesh (1.5 times coarser), yet the global patterns in abundances between these two nets showed relatively high positive correlations (rho > 0.4) for several of the main zooplankton groups (e.g. Total zooplankton, Copepoda, Rhizaria, Eumalacostraca, and Ostracoda + Cladocera; Supplementary Doc. S4). Differences between the WP2 samples and the Régent samples were more marked as the latter was equipped with a mesh 3.4 times larger than the former. Only the abundances of total zooplankton, Cnidaria and Eumalacostraca showed relatively high correlations to the WP2 data.

### Latitudinal patterns of zooplankton size structure

**Figure 3:**
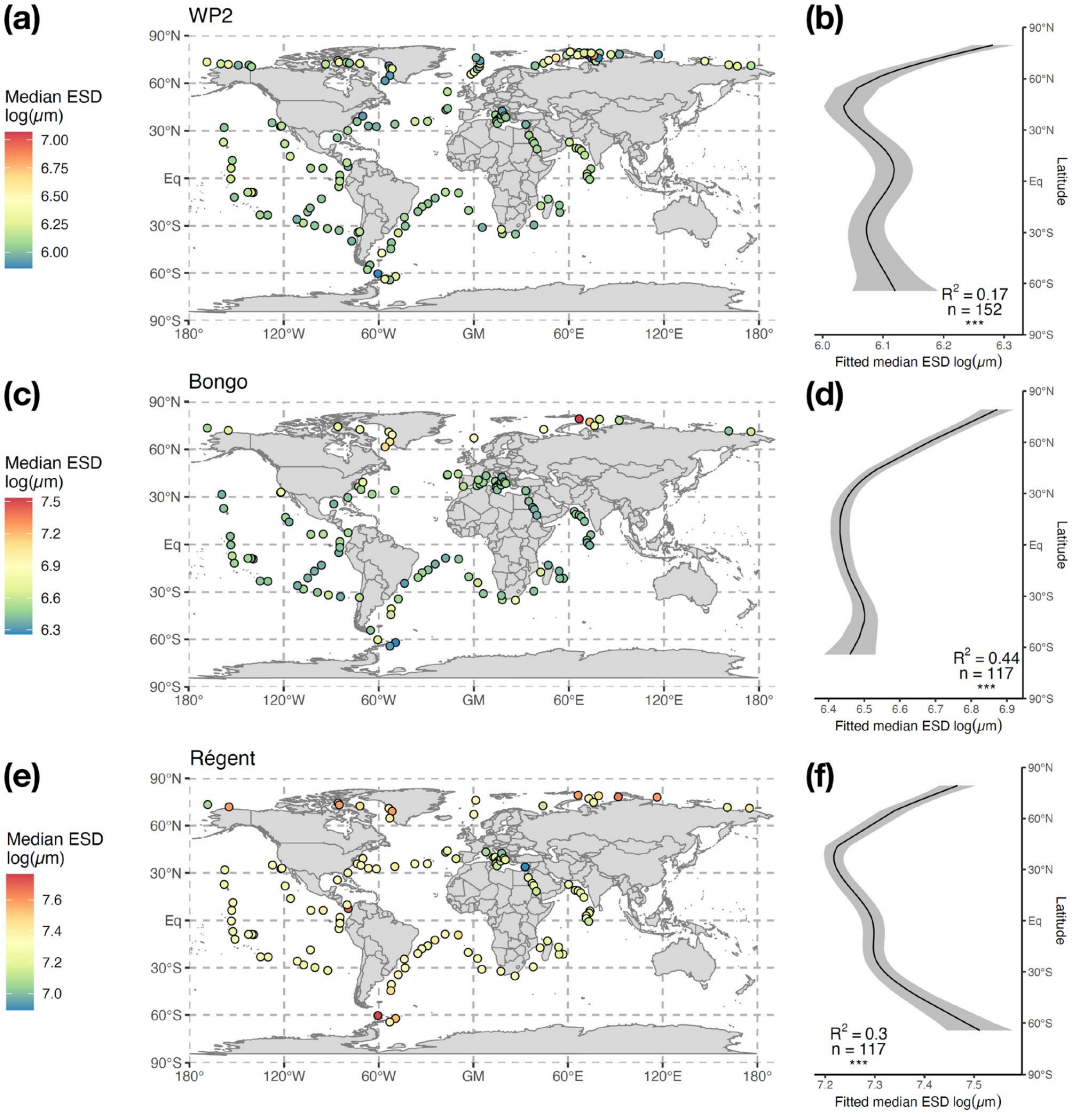
Maps and latitudinal patterns of the logged median Equivalent Spherical Diameter (ESD, µm) observed for Copepoda based on (**a**,**b**) WP2 samples (200 µm mesh), (**c**,**d**) Bongo samples (300 µm mesh) and (**e**,**f**) Régent samples (680 µm mesh). The major and minor axes of the best fitting ellipses were measured for each organism to estimate their ESD. Community-level size structure was determined through the median value of the ESD distribution at individual-level. The solid curves in the right-hand side plots illustrate the prediction from the Generalized Additive Model (GAM) fitting median ESD as a function of latitude. The explanatory power of the GAM (adjusted R2), the number of samples used and the significance of the smooth term (p < 0.001 = ***, p < 0.01 = ***, p < 0.05 = *, p > 0.05 = ns) are reported on the plots. The grey ribbon illustrates the standard error of the prediction. Only the stations where ESD was measured for at least 20 individuals were considered.

Variations in median Equivalent Spherical Diameter (ESD) were explored to examine latitudinal patterns in zooplankton size structure. The most consistent cross-net patterns of median ESD were found for the total zooplankton community, which was driven by the median ESD of calanoid copepods (Fig. 3; see Supplementary Fig. S5 for the other groups). The most prominent feature of the copepod median ESD pattern was a sharp decline from the Arctic to the equator, which was more marked in the Bongo (Fig. 3b) and Régent data (Fig. 3c) than in the WP2 (Fig. 3a). In the southern hemisphere, patterns differed across nets: copepod median ESD sharply increased towards the Southern Ocean according to the Régent net, whereas it showed no variations or a slight decrease according to the WP2 and Bongo samples, respectively. Considering the relatively poor coverage of the Southern Ocean by *Tara* Oceans, these latter patterns should be interpreted with caution. According to the WP2, the net that best sampled the smaller Poecilostomatoida, the latter showed median ESD patterns that were opposite to the Calanoida: their median ESD clearly increased from the poles to the tropics and peaked in the southern hemisphere around 30°S (Supplementary Fig. S5).

Contrary to abundances, a secondary tropical peak in median ESD was not observed for zooplankton (Supplementary Fig. S5). Abundance and median ESD were significantly positively correlated for total zooplankton in the WP2 and Régent data, and for the Copepoda and Calanoida in all nets (Supplementary Table S6).

Among non-copepod groups, the Cnidaria also showed a sharp decrease in median ESD from the Arctic Ocean to the equator in both WP2 and Régent samples (Supplementary Fig. S5). The median ESD of Rhizaria followed the opposite pattern according to the WP2 and Bongo samples as it peaked around 40°N and decreased towards lower latitudes. Our approach did not detect clear latitudinal gradients in median ESD for most of the other zooplankton groups (Supplementary Table S7), either because of insufficient observations or because median ESD is not controlled by factors that vary latitudinally. Therefore, we examined the potential environmental drivers of median ESD variations to help us explain why size structure estimates display less marked latitudinal patterns.

### Relationships with environmental covariates

**Figure 4:**
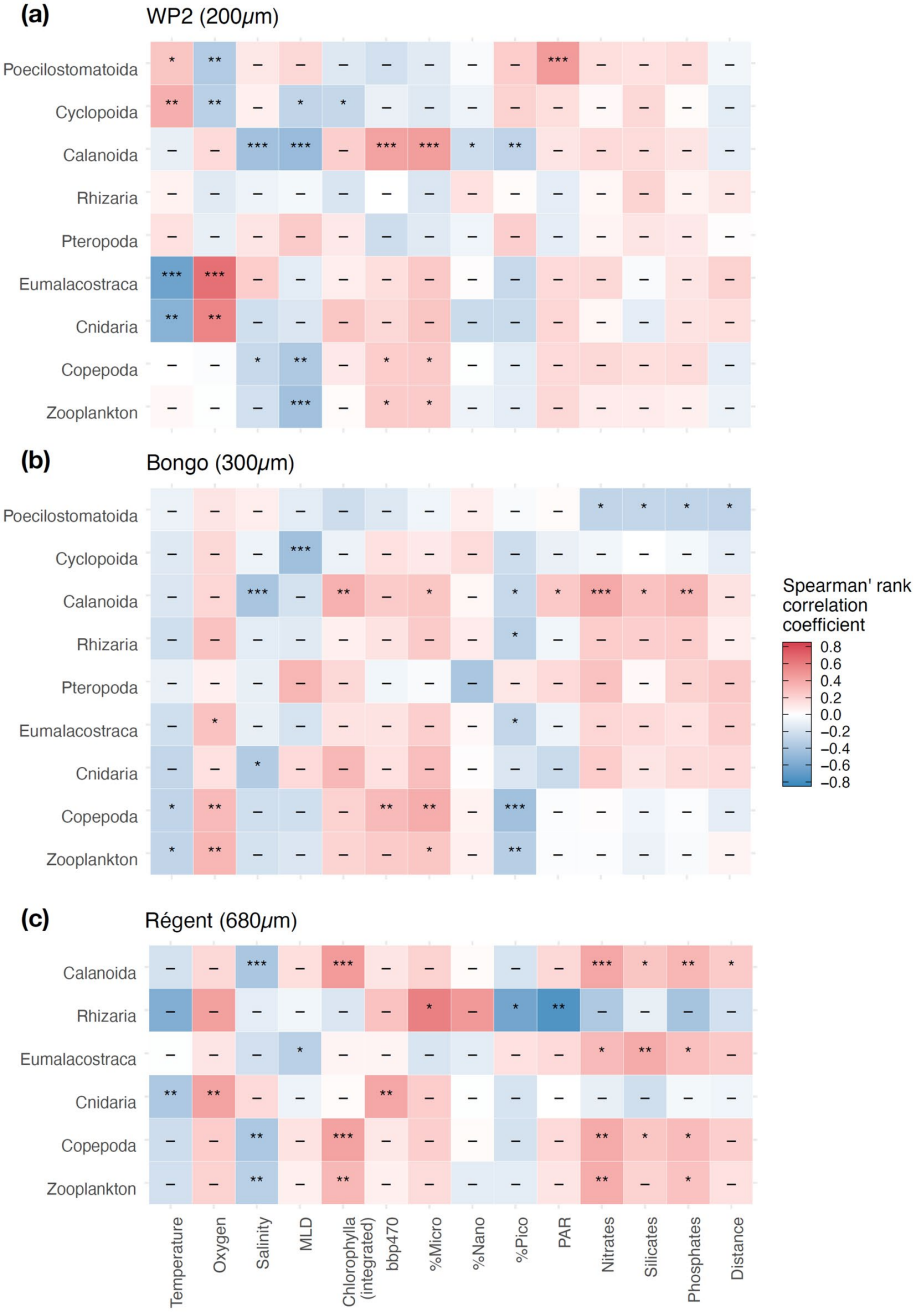
Heatmaps of the Spearman’s rank correlation coefficients computed between the size structure (i.e., logged median Equivalent Spherical Diameter; ESD) of the main zooplankton groups and the selected 14 covariates depicting the environmental conditions in the global surface ocean as sampled by (**a**) WP2 net (200 µm mesh), (**b**) Bongo net (300 µm mesh) and (**c**) Régent net (680 µm). The significance of the Spearman’s rank correlation tests are reported in the tiles (p < 0.001 = ***, p < 0.01 = ***, p < 0.05 = *, p > 0.05 = ns). Only the zooplankton groups displaying significant correlation coefficients for more than one environmental covariate in at least one net parameter are shown (see Supplementary Fig. S8 for all groups). Only the stations where ESD was measured for at least 20 individuals of a group were considered when computing the correlation coefficients. Distance stands for distance to coast (in km).

The strength of the linear covariance between the groups’ abundance, median ESD and environmental covariates was examined through non parametric correlation coefficients (Fig. 4; Supplementary Fig. S8). The median ESD of most zooplankton groups displayed similar significant correlation patterns across nets: the median ESD of total zooplankton, Copepoda, Calanoida, Cnidaria and Eumalacostraca decreased with temperature, salinity and picophytoplankton (%Pico), but increased weakly with oxygen, chlorophyll a, macronutrient concentrations, microphytoplankton (%Micro) and the intensity of particles backscattering (bbp470). Total zooplankton median ESD decreased significantly with Mixed Layer Depth (MLD) only in the WP2 samples (Fig. 4a), a pattern driven by the Calanoida. The median ESD of the Poecilostomatoida increased with temperature, salinity, %Pico and Photosynthetically Active Radiation (PAR). We also found PAR to be the main covariate associated with a lower median ESD of Rhizaria according to the Régent data (Fig. 4c). The Rhizaria showed less significant correlations but differed from the main pattern as their median ESD slightly increased with %Micro and decreases with %Pico and PAR. The only groups displaying a similar pattern were the Tunicata and to a lesser extent the Chaetognatha (Supplementary Fig. S8).

Zooplankton abundances displayed stronger correlation patterns than median ESD (Supplementary Fig. S8) and seem to be more strongly linked to productivity-related covariates (i.e. chlorophyll a, bbp470, %Micro, %Pico and macronutrient concentrations) than physical ones (i.e. temperature and oxygen). The abundance of most groups increased significantly with chlorophyll a, macronutrient concentrations, %Micro and bbp47, but decreased with %Pico. The abundance of some groups presented correlation patterns that departed from the abovementioned trend as they increased with temperature and decreased with oxygen (Supplementary Fig. S8): Rhizaria (WP2 and Régent), Eumalacostraca (Régent mainly), Chaetognatha and Poecilostomatoida (WP2 only).

Nonlinear relationships between median ESD estimates and a subset of environmental covariates were explored through Generalized Additive Models (GAMs, see “Methods”) to identify and rank the drivers of size structure of zooplankton groups (Table 1). In total, 102 GAMs were fitted to median ESD estimates (n = 40 for the WP2 and Bongo data, n = 22 for the Régent; Supplementary Table S7). These GAMs showed reasonable to good fit as the median (± IQR) %Dev was 53.6% (± 33.4%). The GAMs based on the Régent observations displayed significantly higher %Dev (57.9% ± 24.7%) than those based on the WP2 (55.4% ± 31.3%) and Bongo (48.7% ± 34.9%) (Kruskal–Wallis test, Chi^2^ = 143.6, p < 2.2 × 10^−16^). The GAMs including temperature did not show higher %Dev than those including oxygen except with the Régent data but the difference was found to be marginal (Chi^2^ = 19.1, p = 1.3 × 10^−5^). Substantial variations in smooth term rankings were visible across nets and zooplankton groups (Table 1; Supplementary Fig. S9). Oxygen and temperature were the two top-ranking significant covariates, while the remaining eight covariates displayed lower median ranks (Supplementary Fig. S9) though some (e.g., salinity, MLD, chlorophyll a or %Micro) emerged as key covariates for modelling the median ESD of some groups (Table 1).

**Table 1:** Summary of the explanatory power (i.e. % of deviance explained) of the Generalized Additive Models (GAMs) fit to model the global gradients log-transformed Equivalent Spherical Diameter (ESD, µm) as a function of the ten environmental covariates selected, measured for the zooplankton groups and for the plankton nets that sampled enough stations (> 30) and enough individuals (> 20) per group.

Group (median ESD)	Net	First term	Deviance explained (%)	Significant smooth terms (p < 0.05)	Cluster (PAM based on DTW)—only for models with Deviance > 40%
Zooplankton	WP2	Oxygen	0.59	Oxygen, %Micro, %Nano	2
		Temperature	0.54	Temperature, %Micro, %Nano	
	Bongo	Oxygen	0.71	Oxygen, Nitrates, bbp470, %Nano	1
		Temperature	0.72	Temperature, Salinity, Nitrates, bbp470, %Micro, %Nano	
	Régent	Oxygen	0.23	Salinity, bbp470	–
		Temperature	0.23	Salinity, bbp470, %Micro	–
Copepoda	WP2	Oxygen	0.68	Oxygen, PAR, %Micro	2
		Temperature	0.64	Temperature %Micro	
	Bongo	Oxygen	0.89	Oxygen, %Micro, %Nano, Distance to coast	2
		Temperature	0.88	Temperature, %Micro, %Nano, Distance to coast	
	Régent	Oxygen	0.59	Oxygen, Salinity, MLD, PAR	1
		Temperature	0.57	Temperature, Salinity, MLD, PAR	
Rhizaria	WP2	Oxygen	0.76	Oxygen, Salinity, MLD, PAR, Nitrates, bbp470, Chlorophylla, %Nano	3
		Temperature	0.77	Temperature, Salinity, MLD, PAR, Nitrates, bbp470, Chlorophylla, %Nano	
	Bongo	Oxygen	0.47	Oxygen, Salinity	1
		Temperature	0.51	Temperature, Salinity	
Cnidaria	WP2	Oxygen	0.98	Oxygen, Salinity, MLD, PAR, bbp470, %Micro, %Nano, Distance to coast	4
		Temperature	0.96	Temperature, MLD, Nitrates, %Micro, Distance to coast	
	Bongo	Oxygen	0.77	PAR, Nitrates, Chlorophylla, %Nano, Distance to coast	3
		Temperature	0.78	PAR, Nitrates, Chlorophylla, %Nano, Distance to coast	
	Régent	Oxygen	0.22	*Oxygen*	–
		Temperature	0.20	–	–
Tunicata	WP2	Oxygen	0.64	Oxygen, %Micro, %Nano	2
		Temperature	0.59	Temperature, %Micro, %Nano	
	Bongo	Oxygen	0.71	Oxygen, Salinity, MLD, PAR, %Nano	3
		Temperature	0.70	Temperature, PAR, %Nano	
Eumalacostraca	WP2	Oxygen	0.79	Oxygen, Nitrates	2
		Temperature	0.87	Temperature, Nitrates, bbp470, Chlorophylla, %Nano, Distance to coast	
	Bongo	Oxygen	0.54	PAR, Nitrates, %Micro, %Nano	3
		Temperature	0.53	Temperature, PAR, Nitrates, %Nano	
	Régent	Oxygen	0.71	Oxygen, MLD, PAR, Chlorophylla, %Nano	3
		Temperature	0.75	Temperature, MLD, PAR, bbp470, Chlorophylla, %Nano	
Pteropoda	WP2	Oxygen	0.33	Nitrates, bbp470	–
		Temperature	0.33	Nitrates, bbp470	–
Chaetognatha	WP2	Oxygen	0.32	PAR, Chlorophylla	–
		Temperature	0.32	PAR, Chlorophylla	–
	Bongo	Oxygen	0.68	Salinity, bbp470, %Micro, %Nano	3
		Temperature	0.68	Salinity, bbp470, %Micro, %Nano	
	Régent	Oxygen	0.54	Oxygen, Chlorophylla	2
		Temperature	0.60	Temperature, Chlorophylla	
Calanoida	WP2	Oxygen	0.73	Oxygen, PAR, Chlorophylla, %Micro	2
		Temperature	0.73	Temperature, %Micro	
	Bongo	Oxygen	0.50	Oxygen, Nitrates, Chlorophylla	1
		Temperature	0.39	Temperature, Salinity, PAR	
	Régent	Oxygen	0.60	Oxygen, Salinity, MLD, PAR, Nitrates	1
		Temperature	0.58	Temperature, Salinity, MLD, PAR	
Poecilostomatoida	WP2	Oxygen	0.51	MLD, PAR, Distance to coast	3
		Temperature	0.51	MLD, PAR, Distance to coast	
	Bongo	Oxygen	0.39	Oxygen, %Micro, Distance to coast	–
		Temperature	0.35	Temperature, %Micro, Distance to coast	–
Cyclopoida	WP2	Oxygen	0.41	Oxygen, MLD	3
		Temperature	0.41	Temperature, MLD	
	Bongo	Oxygen	0.32	MLD	–
		Temperature	0.32	MLD	–

The significant covariates (p < 0.05) were ranked based on their relative F statistic and are shown. The GAMs displaying a % of deviance explained > 40% were clustered into four groups based on the shape of the smoothing curves of each covariate (Fig. S10).

**Figure 5:**
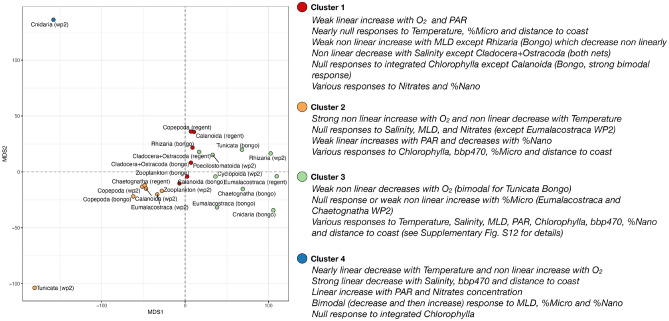
Two dimensional metric dimensional scaling (MDS) plot illustrating the similarity between the responses of the groups’ median ESD to the environmental covariates selected. The smoothing curves from the Generalized Additive Models (GAMs) modelling the global gradients in log-transformed median Equivalent Spherical Diameter (ESD, µm) of the zooplankton groups (estimated for various plankton nets) as a function of ten environmental covariates and displaying a deviance explained > 40%. The smoothing curves were combined into a multivariate data series to compute Dynamic Time Warping (DTW) distances and perform partitioning around medoids (PAM) clustering. This way the GAMs were clustered into four clusters representing combinations of zooplankton groups and plankton nets that exhibit similar median ESD-covariate relationships.

The smoothing curves of the GAMs displaying a %Dev > 50% were extracted to cluster the groups based on the shape of these curves along with each covariate (see Methods). This way, we were able to identify clusters of zooplankton groups displaying similar functional responses to the covariates selected (i.e. zooplankton groups sharing similar drivers of global median ESD), and we could project their similarities in a two dimensional metric dimensional scaling (MDS) space to summarize the main trends. Four clusters were identified (Table 1 and Fig. 5). Cluster 1 comprised six models with a mix of Bongo and Régent observations: the median ESD of total zooplankton and the Calanoida (Bongo), Copepoda and Calanoida (Régent) and Cladocera + Ostracoda (Bongo). This cluster gathered groups whose median ESD showed linear increases with oxygen and PAR and no response to temperature (Supplementary Fig. S10). The smoothing curves modelled for the other covariates were either non-significant or highly variable between groups (Fig. 5; Supplementary Fig. S10). The smoothing curves of the zooplankton WP2 data and its main driving group (i.e. calanoid copepods) were clustered with the Tunicata and Eumalacostraca (WP2), the Copepoda (Bongo) and the Chaetognatha (Régent). Contrary to cluster 1, these groups displayed non linear decreases in median ESD with temperature and relatively strong non liner increases with oxygen. Cluster 3 was the largest as it comprised nine models from various groups and nets: Cyclopoida, Poecilostomatoida and the Rhizaria (all WP2), the Eumalacostraca (Bongo and Régent), the Cladocera + Ostracoda (Régent) and the gelatinous zooplankton (Cnidaria, Tunicata and Chaetognatha) sampled with the Bongo net. Because of the cluster’s larger size, the response curves modelled for these groups were diverse. The main trend was an overall non linear decrease in median ESD with oxygen concentration. Finally, Cluster 4 gathered a single model (Cnidaria, WP2) meaning it displayed an original combination of modelled response curves. The median ESD of the Cnidaria (WP2) decreased linearly with temperature and increased non linearly with oxygen, and it departed from the other groups because of its strong linear decreases with salinity, particles backscattering and distance to coast (Supplementary Fig. S10).

The same approach was applied to investigate the drivers of global abundance patterns (Table 2; Supplementary Table S7). The median ESD-based GAMs display higher %Dev than the abundance-based GAMs (40.7% ± 27.3%; Chi^2^ = 1697.3.6, p < 2.2 × 10^−16^) whatever the net, despite the higher number of observations available for modelling abundances (i.e. 200 GAMs were fitted based on the transformed abundance data). The WP2-based GAMs presented slightly higher %Dev (Chi^2^ = 62.9, p = 2.2 × 10^−14^) than the ones based on the Régent and Bongo observations. The GAMs including temperature displayed a lower %Dev than those including oxygen (37.9 ± 25.8 versus 42.5 ± 27.2; Chi^2^ = 144.1, p < 2.2 × 10^−16^). Contrary to median ESD-GAMs, the inclusion of oxygen instead of temperature substantially increased the %Dev for total zooplankton and Calanoida, and Cyclopoida (Table 2), implying that oxygen could be a stronger driver than temperature for zooplankton abundances. Again, the smooth terms associated with temperature and oxygen emerged as the two most significant terms (Table 2; Supplementary Fig. S9). Substantial variations in smooth terms rankings were observed across nets and groups again (Table 2). However, NO_2_NO_3_ concentrations, chlorophyll a and MLD showed higher significance rankings than in the ESD-based GAMs (Table 2), implying these covariates were more critical to include when modelling zooplankton abundance than size structure.

**Table 2:** Summary of the explanatory power (i.e. % of deviance explained) of the Generalized Additive Models (GAMs) fit to model the global gradients cubic-transformed abundance (ind m^3^) as a function of the ten environmental covariates selected, measured for the zooplankton groups and for the plankton nets that sampled enough stations (> 30) and enough individuals (> 20) per group.

Group (abundance)	Net	First term	Deviance explained (%)	Significant smooth terms (p < 0.05)	Cluster (PAM based on DTW)—only for models with Deviance > 40%
Zooplankton	WP2	Oxygen	0.67	Oxygen, MLD, Nitrates, Chlorophylla, %Nano	4
		Temperature	0.26	Salinity, MLD, Chlorophylla	
	Bongo	Oxygen	0.40	Oxygen, bbp470, %Micro	2
		Temperature	0.38	Temperature, bbp470, %Micro	
	Régent	Oxygen	0.87	Oxygen, PAR, Nitrates, Chlorophylla, %Nano, Distance to coast	1
		Temperature	0.81	Temperature, Nitrates, %Micro, %Nano	
Copepoda	WP2	Oxygen	0.67	Oxygen, MLD, Nitrates, Chlorophylla, %Nano	4
		Temperature	0.30	MLD, Chlorophylla	
	Bongo	Oxygen	0.41	Oxygen, bbp470, %Micro	2
		Temperature	0.38	Temperature, bbp470, %Micro	
	Régent	Oxygen	0.88	Oxygen, Nitrates, Chlorophylla, %Micro, %Nano, Distance to coast	1
		Temperature	0.86	Temperature, Nitrates, %Micro, %Nano, Distance to coast	
Rhizaria	WP2	Oxygen	0.32	Oxygen, Salinity, Nitrates	–
		Temperature	0.32	Temperature, Salinity, Nitrates	–
	Bongo	Oxygen	0.49	Oxygen, Nitrates	2
		Temperature	0.48	Nitrates	
	Régent	Oxygen	0.44	Oxygen, MLD, PAR, bbp470, %Micro	2
		Temperature	0.44	Temperature, PAR, bbp470, %Micro	
Cnidaria	WP2	Oxygen	0.35	Nitrates, Chlorophylla	–
		Temperature	0.35	Nitrates, Chlorophylla	–
	Bongo	Oxygen	0.41	Salinity, MLD, Nitrates	2
		Temperature	0.39	Salinity, MLD, Nitrates	
	Régent	Oxygen	0.21	Distance to coast	–
		Temperature	0.18	Distance to coast	–
Tunicata	WP2	Oxygen	0.40	Oxygen, MLD, Nitrates	3
		Temperature	0.38	Temperature, Nitrates	
	Bongo	Oxygen	0.57	MLD, Nitrates, Chlorophylla	2
		Temperature	0.57	MLD, Nitrates, Chlorophylla	
	Régent	Oxygen	0.22	–	–
		Temperature	0.22	–	–
Eumalacostraca	WP2	Oxygen	0.16	Salinity	–
		Temperature	0.16	Salinity	–
	Bongo	Oxygen	0.24	Nitrates	–
		Temperature	0.24	Nitrates	–
	Régent	Oxygen	0.42	Chlorophylla, %Micro, Distance to coast	2
		Temperature	0.41	Temperature, Chlorophylla	
Pteropoda	WP2	Oxygen	0.33	Oxygen, Nitrates, Chlorophylla, Distance to coast	–
		Temperature	0.32	Temperature, Nitrates, Chlorophylla, Distance to coast	–
	Bongo	Oxygen	0.29	Oxygen, Chlorophylla	–
		Temperature	0.28	Temperature, Chlorophylla	–
	Régent	Oxygen	0.29	%Micro	–
		Temperature	0.27	%Micro	–
Chaetognatha	WP2	Oxygen	0.52	Oxygen, PAR, Chlorophylla, %Nano	2
		Temperature	0.19	Nitrates, %Nano	
	Bongo	Oxygen	0.46	Oxygen, Nitrates, bbp470	2
		Temperature	0.27	bbp470, Chlorophylla	
	Régent	Oxygen	0.43	Nitrates, bbp470, Chlorophylla, %Micro, Distance to coast	2
		Temperature	0.44	Nitrates, bbp470, Chlorophylla, %Micro, Distance to coast	
Calanoida	WP2	Oxygen	0.69	Oxygen, MLD, Chlorophylla, %Micro, %Nano	4
		Temperature	0.35	MLD, Chlorophylla, %Micro	
	Bongo	Oxygen	0.38	Oxygen, bbp470, %Micro	–
		Temperature	0.37	Temperature, bbp470, %Micro	–
	Régent	Oxygen	0.88	Oxygen, Nitrates, Chlorophylla, %Micro, %Nano, Distance to coast	1
		Temperature	0.86	Temperature, Nitrates, %Micro, %Nano	
Poecilostomatoida	WP2	Oxygen	0.53	Oxygen, MLD, Nitrates, Chlorophylla	3
		Temperature	0.51	Temperature, MLD, PAR, Nitrates, Chlorophylla	
	Bongo	Oxygen	0.65	Oxygen, Salinity, MLD, Nitrates	3
		Temperature	0.66	Temperature, Salinity, MLD, Nitrates	
	Régent	Oxygen	0.60	Oxygen, PAR, Distance to coast	2
		Temperature	0.65	Temperature, Salinity, PAR, Distance to coast	
Cyclopoida	WP2	Oxygen	0.64	Oxygen, MLD, PAR, bbp470, Chlorophylla, %Nano	2
		Temperature	0.49	MLD, PAR	
	Bongo	Oxygen	0.45	Salinity, Nitrates, %Nano	2
		Temperature	0.64	Salinity, Nitrates, bbp470, %Nano	
	Régent	Oxygen	0.62	Oxygen, PAR, %Micro	2
		Temperature	0.31	bbp470	

The significant covariates (p < 0.05) were ranked based on their relative F statistic and are shown. The GAMs displaying a % of deviance explained > 40% were clustered into five groups based on the shape of the smoothing curves of each covariate (Fig. S11).

**Figure 6:**
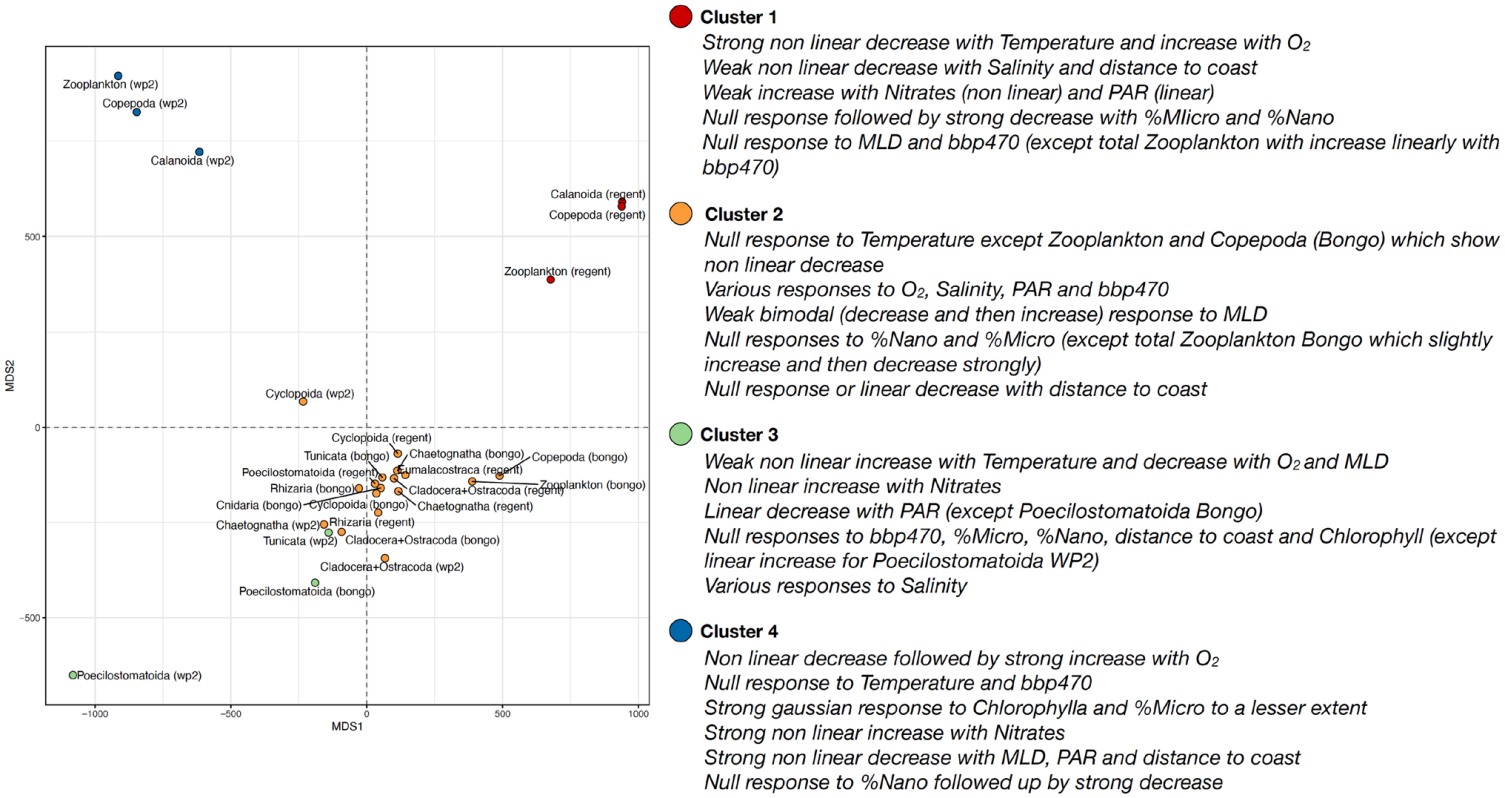
Two dimensional metric dimensional scaling (MDS) plot illustrating the similarity between the responses of the groups’ abundances to the environmental covariates selected. The smoothing curves from the Generalized Additive Models (GAMs) modelling the global gradients in cubic-transformed abundances (ind m^3^) of the zooplankton groups (estimated for various plankton nets) as a function of ten environmental covariates and displaying a deviance explained > 40%. Smoothing curves span a 1–100 scale spanning the range of the covariates measured values. The smoothing curves were combined into a multivariate data series to compute Dynamic Time Warping (DTW) distances and perform partitioning around medoids (PAM) clustering. This way the GAMs were clustered into four clusters that represent combinations of zooplankton groups and plankton nets that exhibit similar abundance-covariate relationships.

Again, the smooth curves of the GAMs displaying a %Dev > 40% were extracted to cluster the zooplankton groups based on the similarity of their responses to the covariates. Four clusters could be identified and these are more clearly delineated than those based on the median ESD response curves as evidenced by the relatively more scaterred positions of the groups in MDS space (Fig. 6; Supplementary Fig. S11). Cluster 1 gathered the smooth curves modelled for total zooplankton, Copepoda and Calanoida based on the Régent data. Their abundances showed: (i) a strong nonlinear decrease with temperature and increase with oxygen concentration, (ii) non linear decreases with salinity and distance to coast, and (iii) slight increases with PAR and NO_2_NO_3_ concentrations. Cluster 2 was the largest clusters as it gathered the responses of diverse range of 17 different models based on various groups and nets. This implies that a relative broad ranges of abundances responses within this cluster (Supplementary Fig. S11), which is why it holds a relatively neutral central position in the MDS space (Fig. 6). Yet, nearly all groups showed null responses in abundances to temperature, except total zooplankton and Copepoda (Bongo data) which showed non linear decreases. Cluster 3 was also a smaller cluster composed of three models only: the Poecilostomatoida (both WP2 and Bongo) and the Tunicata (WP2 only). Contrary to clusters 1 and 2, these were characterized by non linear increases in abundance with temperature and NO_2_NO_3_ concentrations but decreases with oxygen and MLD. This is why these groups are positioned on the negative side of MDS2. Finally, cluster 4 also comprised the same groups as cluster 1 but based on the WP2 abundance estimates instead of the Régent ones. Contrary to the latter, total zooplankton and calanoid copepods here showed null response to temperature. Yet, similar to cluster 1, they also showed strong abundances increase with oxygen concentrations, which explains why both clusters are positioned on the positive side of MDS2 (Fig. 6). This cluster also displayed original strong gaussian responses to chlorophyll a and particles backscattering.

## Discussion

Here, we provide a homogeneous dataset of zooplankton composition and size structure based on individual measurements of body size and document the shape of the relationships between community-level size structure and key environmental drivers on a macroecological scale. We find that zooplankton communities exhibit larger median size and abundance towards the poles and towards the tropical upwelling regions sampled (Supplementary Doc. S12), a pattern that is largely driven by copepods. The higher contributions of the large-bodied grazing Calanidae relative to the smaller omnivorous-carnivorous Cyclopoida and Poecilostomatoida (i.e. Oithonidae, Oncaeidae and Corycaeidae) drives the latitudinal increase in median size towards the poles, in addition to the observed negative scaling of body length with temperature which is in line with the TSR^9,17^. Indeed, our inspection of size structure-environment relationships show that zooplankton size decreases with temperature, salinity, MLD and the contribution of the smallest phytoplankton cells to phytoplankton biomass. Conversely, it increases with concentrations of oxygen, macronutrients, phytoplankton biomass and the contribution of large phytoplankton (e.g. diatoms) to said biomass. Using species body size estimates from the literature, Brun et al.^16^ also found copepod mean body size to increase towards the poles, a pattern driven by a negative temperature-size relationship and a positive relationship between phytoplankton size and zooplankton size. Several explanations for increased body size towards the poles have been proposed, varying from the stimulating effects of temperature on ectotherm metabolism, the synergetic effects of the presence of larger prey, and the availability of oxygen as a function of temperature^2,23^. For metazoan ectotherms, the effects of temperature on somatic and gonad growth seem to be the most robust explanation^24^. The negative correlation between community-level size and temperature might stem from the positive effect of temperature on growth rates. At low latitudes, metabolic rates are higher and life cycles become shorter for the various species composing the community. Consequently community-level median size decreases because of warmer temperatures, and the body surface area to body volume ratio increases^25^. Despite decades of research, it is still uncertain whether the temperature-size rule is an adaptive response to temperature‐related physiological processes (i.e. enzyme activity) or ecological constraints (e.g. food availability, predation and other mortality causes), or a response to biological constraints operating at cellular level such as oxygen supply^12^. Arthropods and rotifers have been shown to reach smaller body sizes in poorly oxygenated waters^23,26^. The potential role of oxygen concentration on the onset of maturation and on size variations remains unclear and is mostly masked by its strong collinearity with surface temperature^12^.

In contrast to the decrease in zooplankton median size and abundance observed towards oligotrophic subtropical gyres, an increase was observed near the equatorial regions where the upwelling regime creates colder and more productive conditions. We found the main groups of the zooplankton communities sampled in the eastern boundary upwellings (EBUS) to display significantly higher abundances relative to communities sampled at comparable latitudes (Supplementary Doc. S12). However, the EBUS do not strongly affect the modelled latitudinal patterns of zooplankton abundance (Supplementary Doc. S12). Yet, the effects of the upwelling regime are more marked for abundances than for size structure. This could be linked to the way we estimated median ESD (e.g. aggregated distributions of body size estimated from particles images) compared to the more direct and less uncertain counting of abundance, or to the fact that fewer stations are available when studying size structure gradients (see “Methods”). Overall, abundances showed correlation patterns with the environmental covariates that are quite similar to median size for the total zooplankton community and its major constituting groups (Calanoida, but also Tunicata, Chaetognatha and Cnidaria). This suggests that zooplankton size structure and abundance respond similarly to environmental drivers. Temperature and/or oxygen concentration were found to be the two main covariates in explaining the quasi-global variations of both size structure and abundance. However, we found productivity-related covariates (i.e. Chlorophyll a, NO_2_NO_3_ concentration, bbp470 and %Micro) to be of higher importance for modelling zooplankton groups abundance. This is an important factor to consider when defining the key parameters to model either zooplankton size or biomass. Our results support the view that temperature and oxygen are more important parameters than the available biomass of photoautotrophs in driving zooplankton community-level and individual-level body size variations^12,17^ and therefore in controlling the expression of physiological traits that scale allometrically (e.g., growth, respiration).

Yet, the abundance of some zooplankton groups (Poecilostomatoida, Rhizaria and Chaetognatha, and Pteropoda to a lesser extent) show correlation patterns that are opposite to the general copepod-driven trend: their abundance actually increases with temperature, PAR and the contribution of small phytoplankton. These groups rely on feeding strategies that are very different from the filter-feeding Calanoida^16,27,28^. For instance, the Poecilostomatoida are cruise-feeding and ambush-feeding copepods displaying a broad omnivorous-carnivorous diet^27–29^. Similarly, chaetognaths are carnivorous ambush-feeders and many pteropods deploy mucus nets for feeding passively on particles fluxes^27^. Therefore, these groups are able to thrive in large phytoplankton-depleted conditions where mortality-risks and competition for food are more pronounced than in phytoplankton-replete conditions thanks to their alternative feeding strategies. If their growth and reproduction are less dependent on phytoplankton biomass while still promoted in warmer conditions, then spatial patterns driven by positive temperature-abundance relationships can emerge. Our results further support the view that zooplankton is not a homogeneous category whose size structure and biomass dynamics can be adequately modelled through a few size classes^1,30^.

We found the median ESD of large protists (i.e. Rhizaria, which mainly comprise Foraminifera and Radiolaria) to increase linearly with %Micro but to decrease with %Pico, PAR, and chlorophyll a to a lesser extent. Contrary to Copepoda, temperature and oxygen did not show clear effect on the size structure of those large protists as their median ESD shows contrasted responses to these two covariates across nets. Large protists abundance increased significantly with temperature, macronutrients concentrations, bbp470 and decreased significantly with oxygen. Therefore, the drivers underlying the patterns of Rhizaria abundance and size structure seem distinct, or even opposite, to those that govern copepod size structure and abundance patterns. Again, this could be ascribed to their notable difference in life strategies. Numerous species of Rhizaria are large single-celled mixotrophic protists that host obligate intracellular microalgal symbionts (photosymbionts^31^). Spinose foraminifera show higher contents of chlorophyll a than the shorter non-spinose species^32^. The efficient photosynthesis performed by photosymbionts, promoted in conditions of higher irradiance and macronutrient concentrations, can lead to oxygen concentrations reaching nearly 200% of the oxygen saturation levels^33,34^, and potentially even more within their cytoplasm. Such high oxygen availability in the protist cells may weaken the usual temperature- and oxygen-driven constraints on their body size. High oxygen concentrations promote the formation of reactive oxygen species (ROS), which could significantly damage cell structures through the oxidation of DNA, cell membranes or proteins. Overproduction of ROS driven by temperature increase is suspected to trigger coral bleaching, either by symbiont expulsion or digestion^35^. Similar reactions may occur within protists^36^. We hypothesize that large protists attempt to prevent ROS accumulation by optimizing the distance between the photosymbionts and themselves. Indeed, most symbiont-bearing Foraminifera tend to display large spinose formation, as a support for the symbiont swarms located further away from the central shell^33,34,37^, but also enhance prey encounter rates^38^. Keeping larger sizes to enhance prey capture and avoid ROS could explain the observed stability in median size and abundance of these organisms in the warmer tropical conditions.

The heterogeneity of sampling strategies between surveys usually hinders global scale plankton studies that require the combination of data from multiple oceanographic cruises. The data collected from the *Tara* Oceans expeditions allow us to examine the in situ properties of plankton communities at a very large spatial scale, thanks to the uniform sampling strategy. However, it should be reminded that the one-time nature of such sampling impedes us from addressing the temporal variations of plankton community size structure across the different provinces studied. In addition, it is also worth to point out that the distribution of the sampling stations are unequal across latitudes (Supplementary Fig. S1). Notwithstanding, the latitudinal patterns we observe for copepod size structure are consistent with those of previous studies that resolved seasonal variations^16,17^, therefore providing some support for the temporal consistency of our results. The correlations we report between abundance, size structure and the environmental variables do not ascertain the ecological and biological processes through which the observed latitudinal patterns emerge. Nonetheless, correlative studies such as ours are key for identifying the major drivers of biological changes and pinpoint further studies to be performed under more controlled conditions that will seek to identify and test the precise biological processes underlying the patterns.

While the level of taxonomic identification of the ZooScan imaging system remains suitable for a size-based community-level study, it does not enable us to depict finer variations in species composition that could be important to further understand the assembly of plankton communities in response to environmental gradients. However, it allowed us to observe large scale patterns and to identify the shape of the relationships between environmental drivers and size structure that would have taken years to depict through non-automated methods. The observed latitudinal patterns in abundance and size structure are relatively consistent across the three nets used but some discrepancies were found (e.g., unidentified Copepoda, Cyclopoida, or Pteropoda; Supplementary Fig. S2). These were likely due to the relative coarse mesh of the Bongo and Régent nets, which underestimated the abundances of most groups (Supplementary Doc. S4). Therefore, these nets could have underestimated the strength of some latitudinal abundance and size structure patterns and their relationships to environmental covariates. Discrepancies between the WP2 data and the two other nets could also stem from differences in sampling depth and net tow, which are known to affect plankton community estimates. The potential effects of these sampling parameters remain difficult to describe here, as only the effects of the mesh size could be evaluated (Supplementary Doc. S4). While the WP2 net was towed vertically from 100 m depth to the surface while the Bongo and Régent nets were towed obliquely from 500 m to the surface. Although these to nets were equipped with coarser meshes, they were towed deeper so they could have captured the deeper living community better^39^. Nonetheless, considering that most of the zooplankton organisms are concentrated in the 0–200 m layer^39^, we are confident that the sampling design of the present *Tara* expeditions adequately captured the macroscale patterns of zooplankton community composition.

Our study follows a trait-based approach to examine the distribution of a “master trait” (i.e. body size) to better investigate how community composition relates to ecosystem functioning^1^. We report quasi-global size-latitude relationships in the size structure of major marine zooplankton groups, as well as their scaling with environmental covariates at the community-level. Larger zooplankton are known to enhance energy fluxes to higher trophic levels and to promote carbon export towards deeper layers^40,41^. Therefore, our observations bring further support to the view that ongoing global climate warming will elicit a decrease in zooplankton size and lower their contribution to the biological carbon pump^41^ as well as to overall metabolic rates^3^. However, fully understanding and predicting such anticipated changes requires a precise parameterization of how environmental conditions impact marine organisms in marine ecosystem models. The representation of plankton diversity in mechanistic marine ecosystem models is improving as the latter may now include from ten^42^ to hundreds of plankton functional types in the case of self-assembling traits-based models^43,44^. Yet vast inter-model discrepancies exist in terms of their parametrization^45^. Models often aim to validate their parameterization using emergent constraints^46,47^. The relationships observed between zooplankton community size structure and environmental covariates, or community biomass per size classes and environmental covariates provide such constraints for model validation and evaluation^45–47^ but also shows that one single parametrization is not sufficient to fully capture the variety of the responses observed among plankton organisms. Therefore, our study allows a more precise parametrization of such models, and thus a more precise estimation of future climatic impact on zooplankton organisms abundance, size and by extension effect on the biological carbon pump. We call for closer collaborations between the fields of macroecology, biology, experimental physiology and adaptation to disentangle the roles of multiple drivers in shaping individual traits and the community-level response of marine ecosystems to current and future cumulative effects of stressors, through cell-to-ecosystem studies^48^.

## Methods

### Sample collection

Zooplankton samples and environmental data were collected at 168 stations across all major oceanic provinces during the *Tara* Oceans expeditions (2009–2013) (Supplementary Fig. S1). Zooplankton was collected with three different types of nets to cover the 200–680 µm size range, encompassing most of the organisms constituting the mesozooplankton. A WP2 net of 200 μm mesh size and 0.57 m^2^ opening was towed vertically or obliquely from 100 m depth to the surface. A Bongo net and a Régent net, of 300 and 680 μm mesh size (0.57 and 1.12 m^2^ opening), respectively, were towed obliquely from 500 m depth to the surface. Samples were preserved with buffered formaldehyde (4%) for later digitization and morphological analyses. The *Tara* Oceans expeditions sampling strategy and methodologies are fully described in Pesant et al.^49^.

### Measurements of environmental covariates

To describe the abiotic habitat associated with each plankton sample, vertical profiles of physical and biogeochemical variables (thereinafter called environmental covariates) were measured by a conductivity temperature depth sensor/rosette (CTD) and Niskin bottles following a published sampling package^50^. A detailed description of each method used as well as all metadata used are available on PANGAEA^51–54^.

Temperature (°C), salinity (psu) and oxygen concentration (µmol kg^−1^) were measured at 10 m depth. Mixed Layer Depth (MLD, m) was estimated based on the 0.03 kg m^−3^ sigma differential density relative to the density at 10 m depth^55^. Chlorophyll a concentration was estimated from vertical CTD casts. The values derived from the fluorescence composite profiles were integrated from 0 to 200 m (or 100 m depending on seafloor depth), using the trapezoidal method. Nutrients concentrations [nitrite/nitrate (NO_2_NO_3_, µmol l^−1^), phosphate (PO_4_, µmol l^−1^) and silicate (SiO_2_, µmol l^−1^)] were determined using segmented flow analysis^56^. For nutrient concentrations, the average of the median values corresponding to each integrated nets samples^53^ was used as it is a better indicator of the overall conditions over the course of a sampling station.

The contribution of the three main phytoplankton size classes to total phytoplankton biomass, %Pico (< 2 µm), %Nano (2–20 µm), and %Micro (> 20 µm) were estimated based on HPLC analysis^57^. The measurements were integrated over the 0–200 water column.

Surface Photosynthetically Active Radiation (PAR, mol quanta m^−2^ day^−1^) was calculated from in situ sensor data, calibrated using factory settings. Surface backscattering coefficient of particles at 470 nm (bbp470, m^−1^) was calculated from in situ sensor data, corrected with in situ measurements in dark conditions. For both PAR and bbp470, we used the median value around the sampling date and location^51–54^.

Among all the contextual metadata provided by the TARA consortium^51–54^, the above-mentioned covariates were selected because: (i) they were the most complete across most sampling stations; (ii) presented the most normal-like distribution and because they were collinear with their alternative versions. Finally, distance to coast (km) was added a posteriori to the suite of covariates to help disentangling coastal samples from the open ocean ones and include this geographical effects in our statistical models. Distance to coast was computed as the shortest Haversine distance to 0 m isobath, on a 15 min resolution. The bathymetric data from the ETOPO1 database (https://ngdc.noaa.gov/mgg/global/global.html) were used and obtained through the *marmap* R package^58^.

### Zooplankton abundance and size estimates

Zooplankton samples were analyzed using the ZooScan imaging system^21^. Zooplankton images classification was performed using an automatic recognition algorithm and validated into taxonomic groups by a posteriori expert inspection. Organisms were classified into coarse taxonomic groups on Ecotaxa^59^, generally at the class or order-level except for copepods which were identified down to the family level whenever possible. For our spatial analyses (see below), 36 taxonomic groups were retained, including total zooplankton, Copepoda, Chaetognatha, Cnidaria, Tunicata (mainly appendicularians, salps and doliolids, which present a range in size, due to change in clade), Eumalacostraca (mainly amphipods, decapods and euphausiids), Rhizaria (mainly foraminifers and radiolarians), Pteropoda and small crustacean grazers (Cladocera, Ostracoda and nauplii larvae). The Copepoda class was then broken down into its main five orders (Calanoida, Cyclopoida, Poecilostomatoida, Harpacticoida and Monstrilloida) which were also broken down to families whenever possible (i.e. to attain n >  = 20 individuals per clade per station; e.g. Oithonidae, Calanidae, Oncaeidae etc.). The last two groups gathered the unidentified Copepoda and the small unidentified Calanoida for which the resolution of the ZooScan did not permit a more precise classification. The list of living organisms identified on Ecotaxa as well as their final taxonomic classification is summarized in Supplementary Table S13. Abundance values were standardized to the number of individuals per m^3^ according to the volume of water filtered. The final mean abundances are the sum of all individuals divided by the volume of water filtered by each net and sample.

The major and minor axes of the best fitting ellipses were measured for each living organism to derive their equivalent spherical diameter (ESD) which is here used as a proxy of body size at the individual-level. Then, the community-level size structure of each of the 36 abovementioned groups was estimated through the median value of the ESD distribution at the individual level. When estimating both abundances and median ESD estimates of a group, all the individuals from the smaller nested groups were accounted for.

### Numerical analyses

Three main steps were carried out: (i) spatial patterns of abundance and size structure were explored; (ii) the strength of their linear relationship with various environmental covariates was examined; and (iii) nonlinear statistical models were fitted based on the groups’ abundances and median ESD and the selected environmental covariates to examine the underlying drivers of global abundance and size structure.

First, the distribution of the groups’ abundance and median ESD were visually inspected for each of the 36 groups and four transformations (square-root, natural log, log to base 10 and cubic) were applied to examine which would you provide the distribution closest to a normal distribution (based on the p-value of Shapiro–Wilk normality tests). As a result, the groups’ abundances were cubic-transformed and the median ESD estimates were log-transformed.

To identify the groups displaying the most compelling global patterns of abundance and size structure, a first set of spatially-explicit generalized additive models (GAMs)^60^ were fitted to the latitude of the sampling stations for each net separately. GAMs are generalized linear models that allow to incorporate flexible nonlinear responses through smoothing functions. GAMs allow us to identify and model nonlinear latitudinal patterns that might emerge because of species-environment relationships. Thin plate regression splines were applied and the smoothing parameters were determined through restricted maximum likelihood (REML) and a Gaussian link function. We insured that at least 30 stations, with each at least 20 individuals were available for fitting the GAMs and thus avoid focusing on groups presenting few observations. Variability in the groups’ abundance may bias the associated median ESD estimates. Some groups of finer taxonomic resolution (i.e. families) may display very low abundances and thus an insufficient number of individuals to derive a robust median ESD estimate from. Therefore, we ensured to consider only on those sampling stations that displayed a sufficient amount of individuals (n ≥ 20) for examining size structure patterns. The adjusted R^2^ of the GAMs, as well as the p-values of the latitude smooth terms, were examined to identify the groups displaying significant latitudinal patterns.

Prior to examining the correlation between the groups’ abundance and median ESD and the selected environmental covariates, the latter were transformed (i.e. square-root, natural log, log to base 10 and cubic) and the normality of the distributions was tested. Macronutrients (NO_2_NO_3_, PO_4_ and SiO_2_) concentrations were cubic-transformed and chlorophyll a concentration was log-transformed. The values of the other covariates were kept as is. Spearman’s rank correlation coefficients (ρ) were computed between the groups’ abundance/median ESD and all covariates to examine the strength of their linear relationships and identify the main drivers of the spatial patterns. Then, the shape of specific abundance-environment and size-environment relationships were modelled through GAMs. A prior selection of covariates was carried out to discard those that would be too collinear^61^. Covariates collinearity is a sensitive issue when modelling biotic-abiotic relationships through regressive models as it may inflate parameters and errors estimates^61^. For each net data, pairwise Spearman’s correlation coefficients (ρ) were computed between covariates (Supplementary Fig. S14). When a pair of covariates displayed a |ρ|≥ 0.7, the covariate displaying the distribution closest to normality and the least amount of missing values was retained. As a result, %Pico was discarded to the advantage of %Micro (ρ = 0.85), and PO_4_ and SiO_2_ concentrations were discarded to the advantage of NO_2_NO_3_ concentration (ρ = 0.95 and ρ = 0.87, respectively). NO_2_NO_3_ was thus used to represent gradients in macronutrients concentration. Temperature and dissolved oxygen concentration also displayed high collinearity (ρ = − 0.95). Yet, we wanted to assess how important both these covariates could be in explaining abundance and size structure patterns. Therefore we ran the subsequent analyses by accounting for temperature and O_2_ but separately.

Using the groups transformed abundance and median ESD as response variables, we fitted GAMs for each net data using the following ten covariates: temperature, salinity, MLD, PAR, NO_2_NO_3_, chlorophyll a, bbp470, %Micro, %Nano and distance to coast. A second set of GAMs was trained by replacing temperature by oxygen. Again, thin plate regression splines were applied and the parameters were determined through REML and a Gaussian link function. The dimensions of the basis of the smooth terms were adjusted by dividing the number of available observations by the number of covariates. Extra penalties were added to each smoothing term so the parameter estimation can completely remove terms estimated as insignificant. Model terms were then selected by backwards removal of insignificant variables. The percentage of explained deviance (%Dev) and the adjusted R^2^ of the GAMs were retrieved to evaluate their performance. For each GAM, covariates significance was ranked according to their relative F statistic (Supplementary Fig. S9). We tested for significant variations in %Dev across net data or covariates sets through non-parametric variance analyses (Kruskal–Wallis test) followed by posthoc pairwise comparisons using Dunn’s test and Bonferroni’s method for adjusting p-values. Only those stations displaying more than 20 individuals when modelling the groups’ size structure estimates were considered. The significant smooth terms (p-values < 0.05) were identified and their smoothing function and standard error estimates were plotted on the scale of the corresponding covariate (covariate-specific smooth curve).

The covariate-specific smooth curves of the GAMs fitted on median ESD estimates displaying a %Dev ≥ 50% were then used to cluster the zooplankton groups based on the shapes of the modelled smooth curves. This way, we identified groups that respond similarly to environmental gradients. In short, the smooth curves were treated as independent data series and we used Dynamic Time Warping (DTW)^62^ to compare them to each other. DTW is an algorithm that tries to find the optimum warping path between two univariate or multivariate data series. DTW stretches the data series locally to have one match the other(s) as much as possible. Then, the Euclidean distance between the data series is computed by summing the distances of the aligned data points. The modelled smooth curves were projected on a scale of 1 to 100 values scaling the range observed for each covariate. All the covariate-specific smooth curves were out together in a list that can be assimilated to a multivariate data series even in lengths for each retained GAM. DTW distances were then computed and partitioning around medoids (PAM) clustering^63^ was performed to cluster the GAMs (i.e. a zooplankton group + a net type) into two to ten clusters. Five different indices (Calinski-Harabasz, Dunn’s, Silhouettes, classic and modified Davies Bouldin indices) were examined to choose the optimal number of cluster. Four clusters were retained for the median-ESD based GAMs, and four for the abundance-based ones (Supplementary Fig. S15). Hierarchical clustering approaches were also examined but they yielded unclear performance indices so PAM was preferred. Every modelled covariate-specific smooth curves used for the DTW clustering are reported in Supplementary Figs. S10 and S11 for the median ESD responses and the abundance models, respectively. To summarize such large amount of information and illustrate the similarities between the zooplankton groups, the inter-group distance matrix issued from the DTW algorithm was projected onto to a two dimensional space based on classical multidimensional scaling.

The analyses were performed with the R v3.5.2^64^ environment and with MATLAB R2017a. All maps presented were plotted in R v3.5.2. The main packages used for data analyses and plotting were *tidyverse*^65^ and *HH*^66^ and *FactoMineR*^67^. GAMs were built using the *mgcv*^60^ package. The partitional clustering of the zooplankton groups based on the shape of the smoothing curves issued from the GAMs was performed using the *dtwclust*^68^ package.

## Supplementary Information

Supplementary Information 1.

Supplementary Information 2.

Supplementary Information 3.

Supplementary Information 4.

Supplementary Information 5.

## Data Availability

Median ESD and abundance values by zooplankton groups are available at 10.17632/nwvjwccgvh.1. Zooplankton imaging datasets from the *Tara* Oceans expeditions are available through the collaborative web Ecotaxa application and repository under the addresses: https://ecotaxa.obs-vlfr.fr/prj/377, https://ecotaxa.obs-vlfr.fr/prj/2245, https://ecotaxa.obs-vlfr.fr/prj/378 for the WP2 net; https://ecotaxa.obs-vlfr.fr/prj/397, https://ecotaxa.obs-vlfr.fr/prj/398, https://ecotaxa.obs-vlfr.fr/prj/395 for the Bongo net; https://ecotaxa.obs-vlfr.fr/prj/415, https://ecotaxa.obs-vlfr.fr/prj/409, https://ecotaxa.obs-vlfr.fr/prj/408, https://ecotaxa.obs-vlfr.fr/prj/411, https://ecotaxa.obs-vlfr.fr/prj/412 for the Régent net. Contextual data from the *Tara* Oceans expedition, including those that are newly released from the Arctic Ocean, are available at https://doi.org/10.1594/PANGAEA.875582.
