# Author Correction: Macroscale patterns of oceanic zooplankton composition and size structure

**DOI:** 10.1038/s41598-021-99772-1

**Published:** 2021-10-06

**Authors:** Manoela C. Brandão, Fabio Benedetti, Séverine Martini, Yawouvi Dodji Soviadan, Jean-Olivier Irisson, Jean-Baptiste Romagnan, Amanda Elineau, Corinne Desnos, Laëtitia Jalabert, Andrea S. Freire, Marc Picheral, Lionel Guidi, Gabriel Gorsky, Chris Bowler, Lee Karp-Boss, Nicolas Henry, Colomban de Vargas, Matthew B. Sullivan, Tara Oceans Consortium Coordinators, Silvia G. Acinas, Marcel Babin, Peer Bork, Emmanuel Boss, Chris Bowler, Guy Cochrane, Colomban de Vargas, Gabriel Gorsky, Lionel Guidi, Nigel Grimsley, Pascal Hingamp, Daniele Iudicone, Olivier Jaillon, Stefanie Kandels, Lee Karp-Boss, Eric Karsenti, Fabrice Not, Hiroyuki Ogata, Nicole Poulton, Stephane Pesant, Jeroen Raes, Christian Sardet, Sabrina Speich, Lars Stemmann, Matthew B. Sullivan, Shinichi Sunagawa, Patrick Wincker, Lars Stemmann, Fabien Lombard

**Affiliations:** 1 Sorbonne Université, CNRS, Laboratoire d’Océanographie de Villefranche, 06230 Villefranche-sur-mer, France; 2 Ifremer, Centre Bretagne, Unité Dynamiques des Ecosystèmes Côtiers, 29280 Plouzané, France; 3 ETH Zürich, Institute of Biogeochemistry and Pollutant Dynamics, 8092 Zürich, Switzerland; 4 Aix Marseille Univ., Université de Toulon, CNRS, IRD, MIO UM 110, 13288 Marseille, France; 5 Ifremer, Centre Atlantique, Unité Ecologie et Modèles Pour l’Halieutique, 44311 Nantes, France; 6 Departamento de Ecologia e Zoologia, Universidade Federal de Santa Catarina, Florianópolis, 88010970 Brazil; 7 Institut de Biologie de l’École Normale Supérieure (IBENS), CNRS, INSERM, PSL Université Paris, 75005 Paris, France; 8 Research Federation for the Study of Global Ocean Systems Ecology and Evolution, FR2022/Tara Oceans GOSEE, 75016 Paris, France; 9 School of Marine Sciences, University of Maine, Orono, 04469 USA; 10 Sorbonne Université, CNRS, Station Biologique de Roscoff, AD2M, UMR 7144, 29680 Roscoff, France; 11 Department of Microbiology and Civil, Environmental, and Geodetic Engineering, The Ohio State University, Columbus, 43214 USA; 12 Institut Universitaire de France, 75231 Paris, France; 13 Institute of Marine Sciences (ICM) – CSIC, Pg. Marítim de la Barceloneta, 37-49, 08003 Barcelona, Spain; 14 Takuvik Joint International Laboratory (UMI3376), Université Laval (Canada) – CNRS (France), Université Laval, Québec, QC G1V 0A6 Canada; 15 Structural and Computational Biology, European Molecular Biology Laboratory, Meyerhofstrasse 1, 69117 Heidelberg, Germany; 16 European Molecular Biology Laboratory, European Bioinformatics Institute (EMBL-EBI), Wellcome Trust Genome Campus, Hinxton, Cambridge, UK; 17 CNRS Biologie Intégrative Des Organismes Marins (BIOM), UMR7232, 1 avenue Pierre Fabre, 66650 Banyuls-sur-Mer, France; 18 Sorbonne Université, Observatoire Océanologique de Banyuls-Sur-Mer, 1 avenue Pierre Fabre, 66650 Banyuls-sur-Mer, France; 19 Stazione Zoologica Anton Dohrn, Villa Comunale, 80121 Naples, Italy; 20 Génomique Métabolique, Genoscope, Institut de Biologie François Jacob, Commissariat à l’Énergie Atomique (CEA), CNRS, Université Évry, Université Paris-Saclay, Évry, France; 21 Directors’ Research, European Molecular Biology Laboratory, Meyerhofstrasse 1, 69117 Heidelberg, Germany; 22 Institute for Chemical Research, Kyoto University, Gokasho, Uji, Kyoto, 611-001 Japan; 23 Bigelow Laboratory for Ocean Sciences, East Boothbay, ME 04544 USA; 24 PANGAEA, Data Publisher for Earth and Environmental Science, University of Bremen, Bremen, Germany; 25 MARUM, Center for Marine Environmental Sciences, University of Bremen, Bremen, Germany; 26 Department of Microbiology and Immunology, Rega Institute, KU Leuven, Herestraat 49, 3000 Leuven, Belgium; 27 Center for the Biology of Disease, VIB, Herestraat 49, 3000 Leuven, Belgium; 28 Department of Applied Biological Sciences, Vrije Universiteit Brussel, Pleinlaan 2, 1050 Brussels, Belgium; 29 Sorbonne Université, CNRS, UMR 7009 Biodev, Observatoire Océanologique, 06230 Villefranche-sur-mer, France; 30 Department of Geosciences, Laboratoire de Météorologie Dynamique (LMD), Ecole Normale Supérieure, 24 rue Lhomond, 75231 Paris Cedex 05, France; 31 Laboratoire de Physique des Océans, UBO-IUEM, Place Copernic, 29820 Plouzané, France; 32 Department of Biology, Institute of Microbiology and Swiss Institute of Bioinformatics, ETH Zürich, Vladimir-Prelog-Weg 4, 8093 Zürich, Switzerland

Correction to: *Scientific Reports* 10.1038/s41598-021-94615-5, published online 03 August 2021

The original version of this Article contained an error in the Acknowledgements section, where,

“F.B. received support from ETH Zürich and from the European Union’s Horizon 2020 research and innovation program under grant agreement n°SEP-210591007.”

now reads:

“F.B. received support from ETH Zürich. This project has received funding from the European Union’s Horizon 2020 research and innovation programme under grant agreement No 862923. This output reflects only the author’s view, and the European Union cannot be held responsible for any use that may be made of the information contained therein.”

The original Article has been corrected.

