# Supplementary material for: Macroscale patterns of oceanic zooplankton composition and size structure
Source: Sci Rep. 2021 Aug 3;11:15714. doi: 10.1038/s41598-021-94615-5 (PMC8333327; doi:10.1038/s41598-021-94615-5)
Supplement: Supplementary file 4 — Supplementary Information 4. [file 41598_2021_94615_MOESM4_ESM.pdf]

Macroscale patterns of oceanic zooplankton composition and size structure

Manoela C. Brandão[1,2,+,*], Fabio Benedetti[3,+,*], Séverine Martini[4], Yawouvi Dodji Soviadan[1], Jean-Olivier Irisson[1], Jean-Baptiste Romagnan[5], Amanda Elineau[1], Corinne Desnos[1], Laëtitia Jalabert[1], Andrea S. Freire[6], Marc Picheral[1], Lionel Guidi[1], Gabriel Gorsky[1], Chris Bowler[7,8], Lee Karp-Boss[9], Nicolas Henry[8,10], Colomban de Vargas[8,10], Matthew B. Sullivan[11], *Tara* Oceans Consortium Coordinators[‡], Lars Stemmann[1,8], and Fabien Lombard[1,8,12]

[1]Sorbonne Université, CNRS, Laboratoire d'Océanographie de Villefranche, Villefranche-sur-mer, 06230, France
[2]Ifremer, Centre Bretagne, Unité Dynamiques des Ecosystèmes Côtiers, Plouzané, 29280, France
[3]ETH Zürich, Institute of Biogeochemistry and Pollutant Dynamics, Zürich, 8092, Switzerland
[4]Aix Marseille Univ., Université de Toulon, CNRS, IRD, MIO UM 110, Marseille, 13288, France
[5]Ifremer, Centre Atlantique, Unité Ecologie et Modèles pour l'Halieutique, Nantes, 44311, France
[6]Universidade Federal de Santa Catarina, Departamento de Ecologia e Zoologia, Florianópolis, 88010970, Brazil
[7]Institut de biologie de l'École normale supérieure (IBENS), CNRS, INSERM, PSL Université Paris, Paris, 75005, France
[8]Research Federation for the study of Global Ocean Systems Ecology and Evolution, FR2022/*Tara* Oceans GOSEE, Paris, 75016, France
[9]University of Maine, School of Marine Sciences, Orono, 04469, USA
[10]Sorbonne Université, CNRS, Station Biologique de Roscoff, AD2M, UMR 7144, Roscoff, 29680, France
[11]The Ohio State University, Department of Microbiology and Civil, Environmental, and Geodetic Engineering, Columbus, 43214, USA
[12]Institut Universitaire de France, Paris, 75231, France

[+]these authors contributed equally to this work
[*]manoelacb1@gmail.com; fabio.benedetti@usys.ethz.ch
[‡]*Tara* Oceans Consortium Coordinators and affiliations are listed at the end of this manuscript

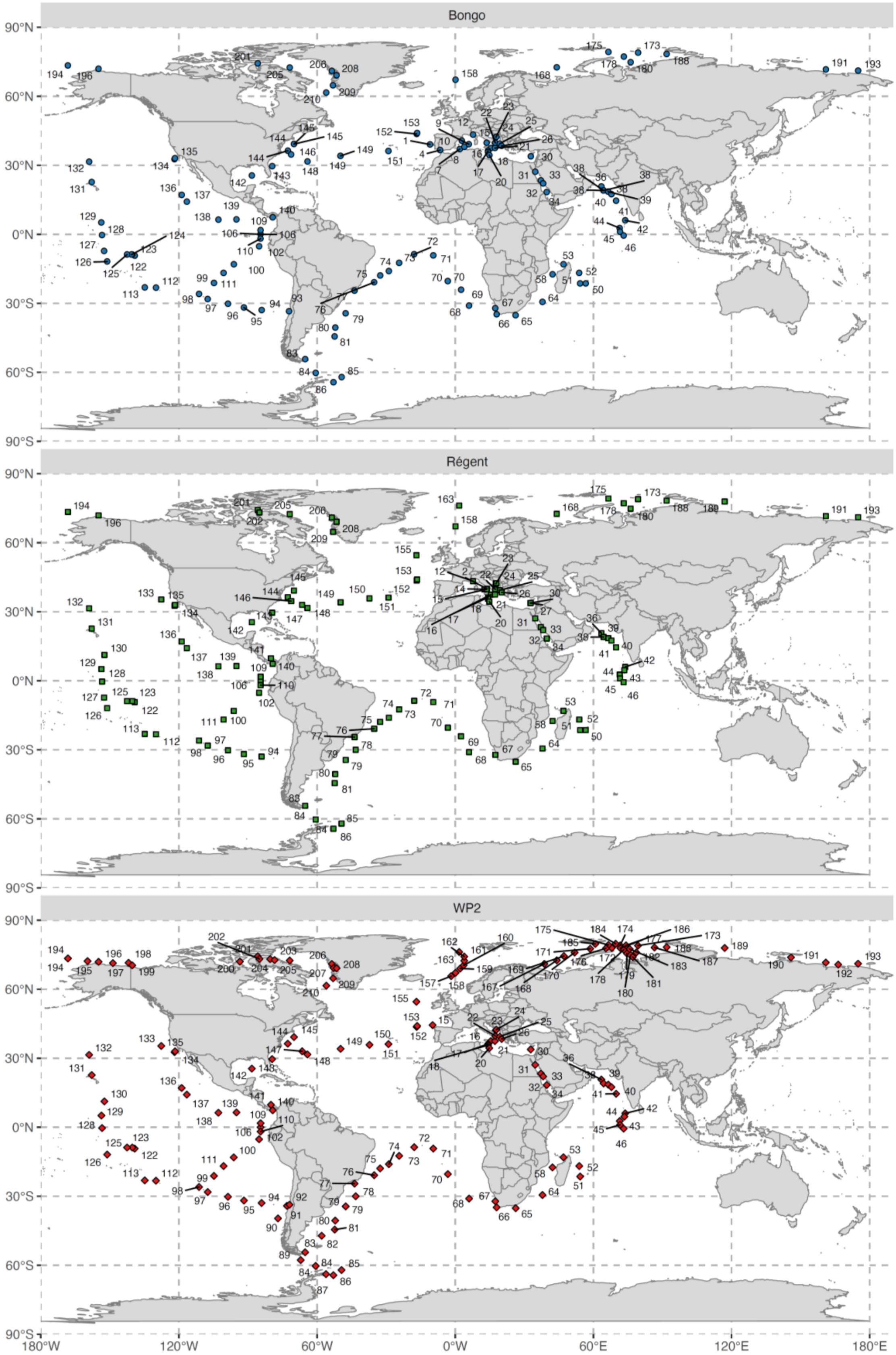

Supplementary Figure S1: Location of the sampling stations of the *Tara* Oceans expedition used in the present study discriminated per plankton net.

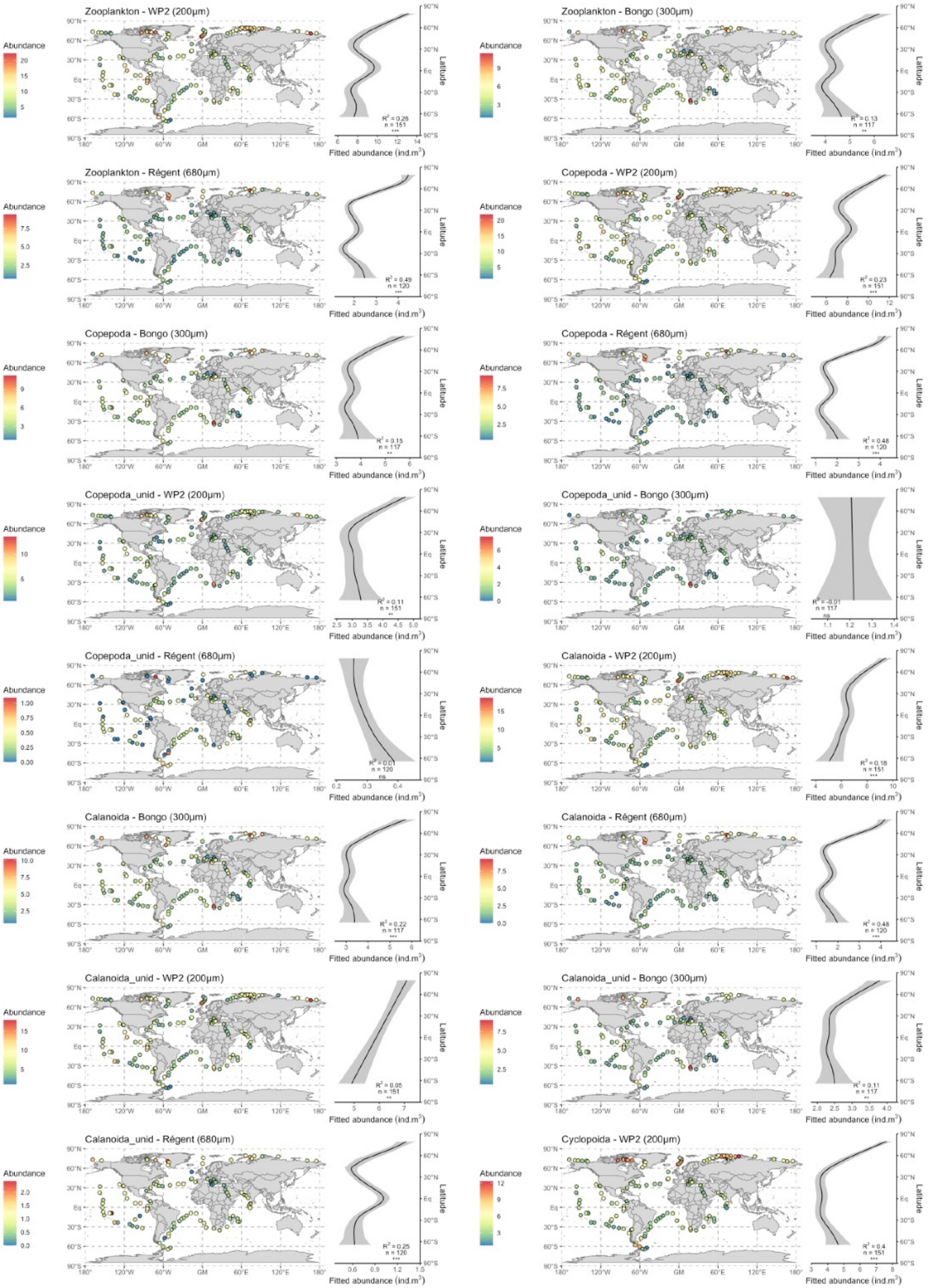

Zooplankton - WP2 (200µm)
Zooplankton - Bongo (300µm)
Zooplankton - Régent (680µm)
Copepoda - WP2 (200µm)
Copepoda - Bongo (300µm)
Copepoda - Régent (680µm)
Copepoda_unid - WP2 (200µm)
Copepoda_unid - Bongo (300µm)
Copepoda_unid - Régent (680µm)
Calanoida - WP2 (200µm)
Calanoida - Bongo (300µm)
Calanoida - Régent (680µm)
Calanoida_unid - WP2 (200µm)
Calanoida_unid - Bongo (300µm)
Calanoida_unid - Régent (680µm)
Cyclopoida - WP2 (200µm)
Abundance
Latitude
Fitted abundance (ind.m3)

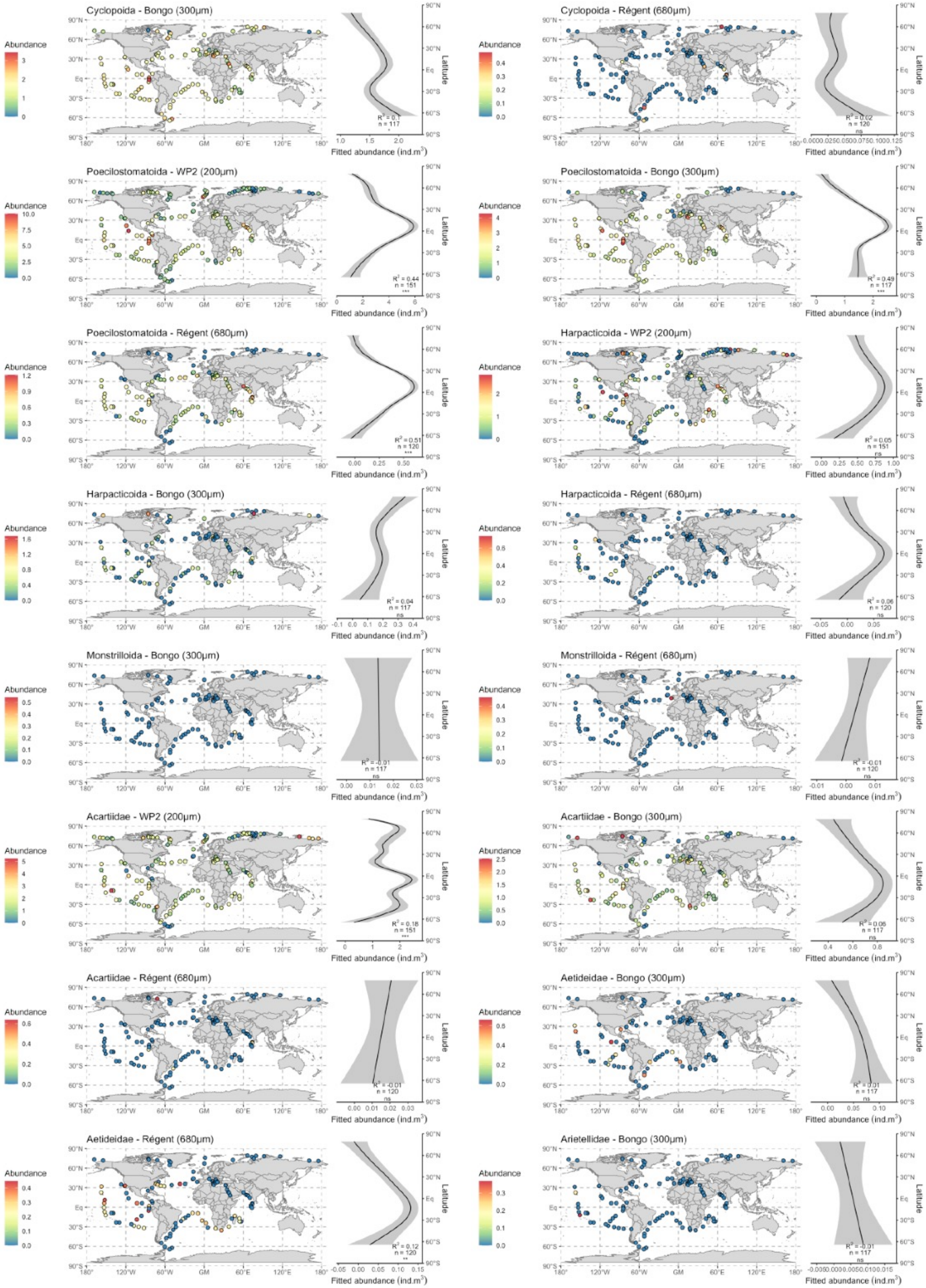
Cyclopoida - Bongo (300µm)
R² = 0.1
n = 117
Cyclopoida - Régent (680µm)
R² = 0.02
n = 120
ns
Poecilostomatoida - WP2 (200µm)
R² = 0.44
n = 151
***
Poecilostomatoida - Bongo (300µm)
R² = 0.49
n = 117
***
Poecilostomatoida - Régent (680µm)
R² = 0.51
n = 120
***
Harpacticoida - WP2 (200µm)
R² = 0.05
n = 151
ns
Harpacticoida - Bongo (300µm)
R² = 0.04
n = 117
ns
Harpacticoida - Régent (680µm)
R² = 0.06
n = 120
ns
Monstrilloida - Bongo (300µm)
R² = -0.01
n = 117
ns
Monstrilloida - Régent (680µm)
R² = -0.01
n = 120
ns
Acartiidae - WP2 (200µm)
R² = 0.18
n = 151
***
Acartiidae - Bongo (300µm)
R² = 0.06
n = 117
ns
Acartiidae - Régent (680µm)
R² = -0.01
n = 120
ns
Aetideidae - Bongo (300µm)
R² = 0.01
n = 117
ns
Aetideidae - Régent (680µm)
R² = 0.12
n = 120
**
Arietellidae - Bongo (300µm)
R² = -0.01
n = 117
ns
Abundance
Latitude
Fitted abundance (ind.m³)

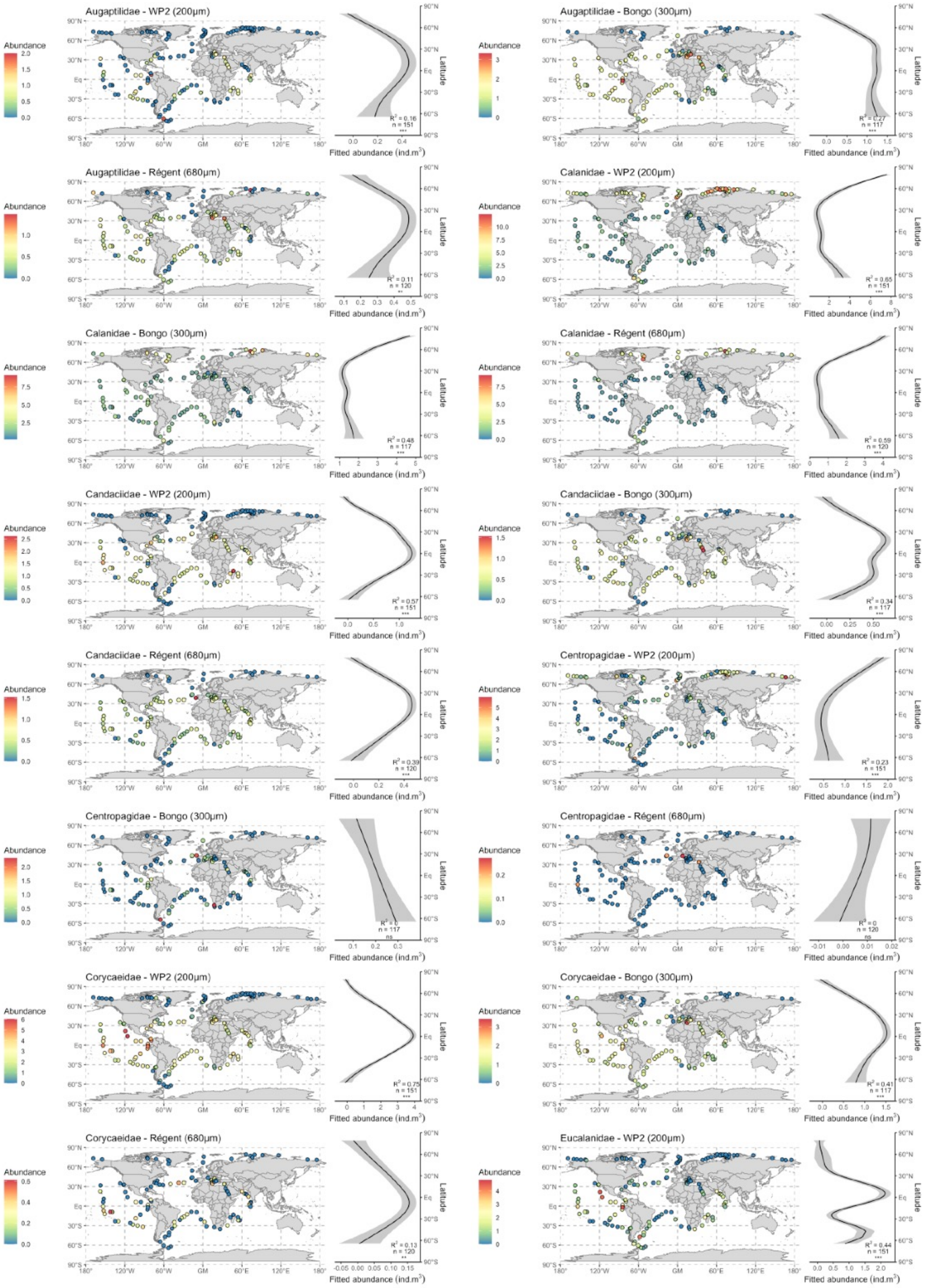
Augaptilidae - WP2 (200µm)
R² = 0.16
n = 151
Augaptilidae - Bongo (300µm)
R² = 0.27
n = 117
Augaptilidae - Régent (680µm)
R² = 0.11
n = 120
Calanidae - WP2 (200µm)
R² = 0.65
n = 151
Calanidae - Bongo (300µm)
R² = 0.48
n = 117
Calanidae - Régent (680µm)
R² = 0.59
n = 120
Candaciidae - WP2 (200µm)
R² = 0.57
n = 151
Candaciidae - Bongo (300µm)
R² = 0.34
n = 117
Candaciidae - Régent (680µm)
R² = 0.39
n = 120
Centropagidae - WP2 (200µm)
R² = 0.23
n = 151
Centropagidae - Bongo (300µm)
R² = 0
n = 117
ns
Centropagidae - Régent (680µm)
R² = 0
n = 120
ns
Corycaeidae - WP2 (200µm)
R² = 0.75
n = 151
Corycaeidae - Bongo (300µm)
R² = 0.41
n = 117
Corycaeidae - Régent (680µm)
R² = 0.13
n = 120
Eucalanidae - WP2 (200µm)
R² = 0.44
n = 151
Abundance
Latitude
Fitted abundance (ind.m3)

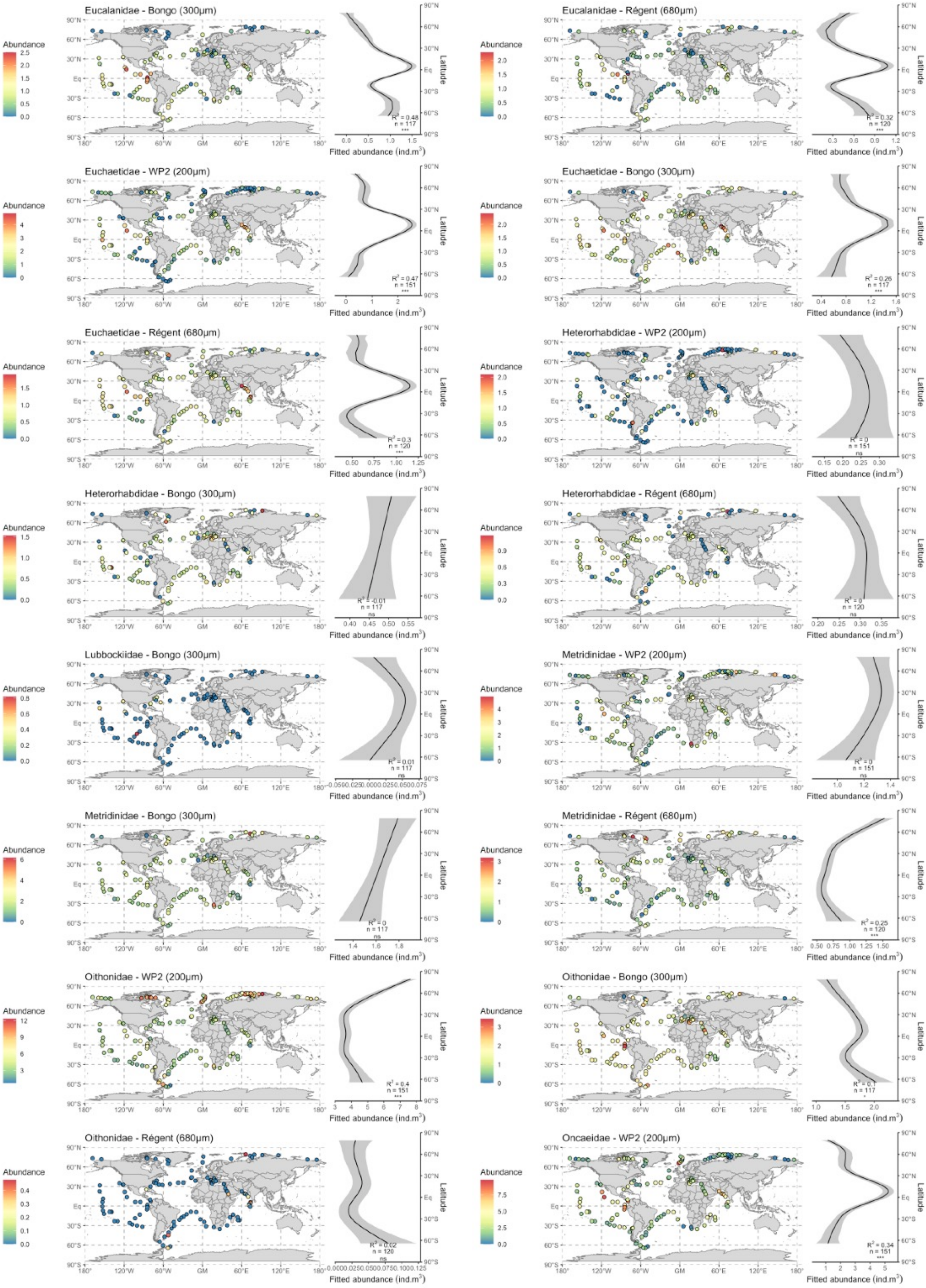

Eucalanidae - Bongo (300µm)
Eucalanidae - Régent (680µm)
Euchaetidae - WP2 (200µm)
Euchaetidae - Bongo (300µm)
Euchaetidae - Régent (680µm)
Heterorhabdidae - WP2 (200µm)
Heterorhabdidae - Bongo (300µm)
Heterorhabdidae - Régent (680µm)
Lubbockiidae - Bongo (300µm)
Metridinidae - WP2 (200µm)
Metridinidae - Bongo (300µm)
Metridinidae - Régent (680µm)
Oithonidae - WP2 (200µm)
Oithonidae - Bongo (300µm)
Oithonidae - Régent (680µm)
Oncaeidae - WP2 (200µm)
Abundance
Latitude
Fitted abundance (ind.m3)

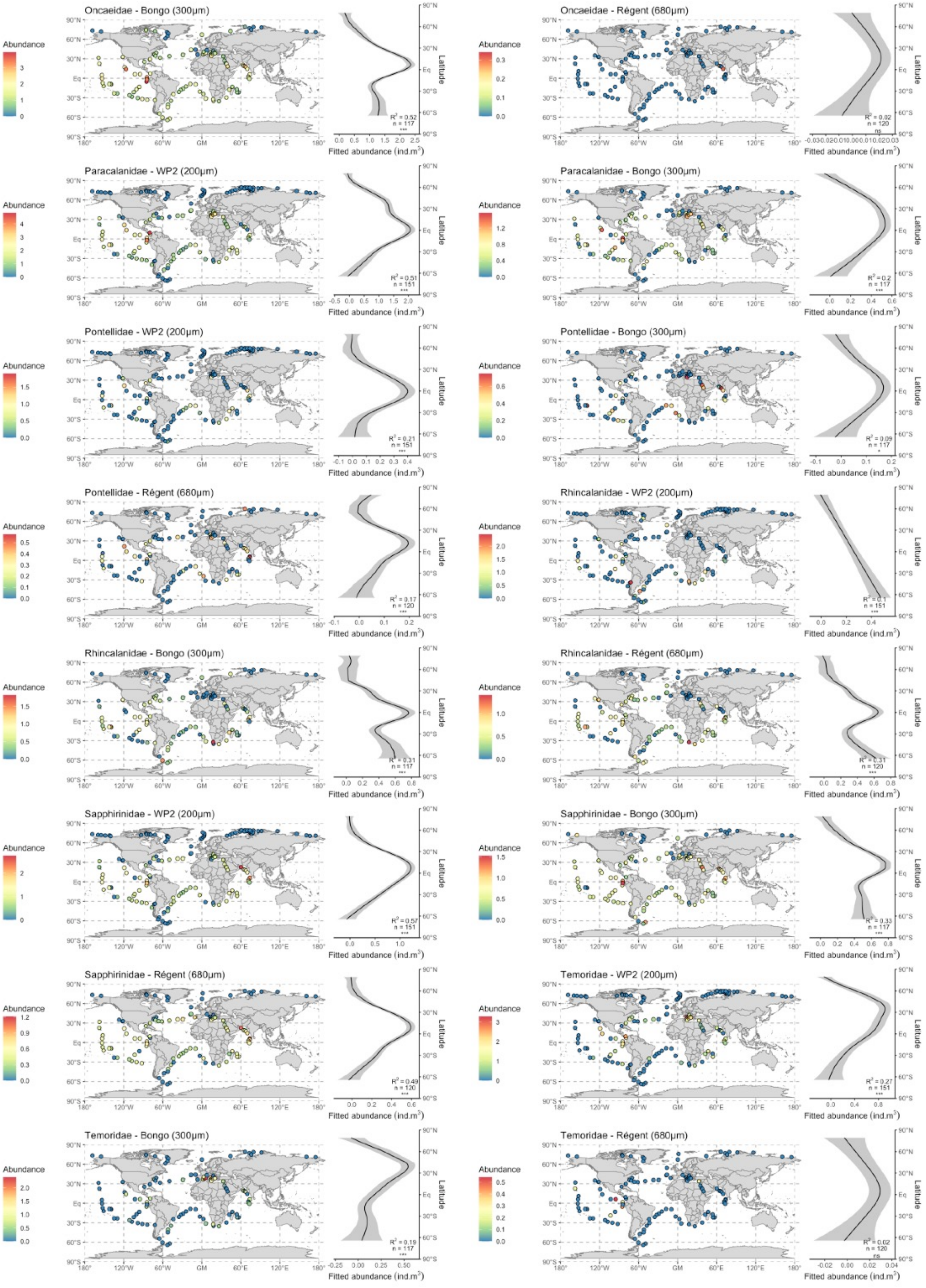

Oncaeidae - Bongo (300µm)
R² = 0.52
n = 117
***
Oncaeidae - Régent (680µm)
R² = 0.02
n = 120
ns
Paracalanidae - WP2 (200µm)
R² = 0.51
n = 151
***
Paracalanidae - Bongo (300µm)
R² = 0.2
n = 117
***
Pontellidae - WP2 (200µm)
R² = 0.21
n = 151
***
Pontellidae - Bongo (300µm)
R² = 0.09
n = 117
*
Pontellidae - Régent (680µm)
R² = 0.17
n = 120
***
Rhincalanidae - WP2 (200µm)
R² = 0.1
n = 151
***
Rhincalanidae - Bongo (300µm)
R² = 0.31
n = 117
***
Rhincalanidae - Régent (680µm)
R² = 0.31
n = 120
***
Sapphirinidae - WP2 (200µm)
R² = 0.57
n = 151
***
Sapphirinidae - Bongo (300µm)
R² = 0.33
n = 117
***
Sapphirinidae - Régent (680µm)
R² = 0.49
n = 120
***
Temoridae - WP2 (200µm)
R² = 0.27
n = 151
***
Temoridae - Bongo (300µm)
R² = 0.19
n = 117
***
Temoridae - Régent (680µm)
R² = 0.02
n = 120
ns
Abundance
Latitude
Fitted abundance (ind.m3)

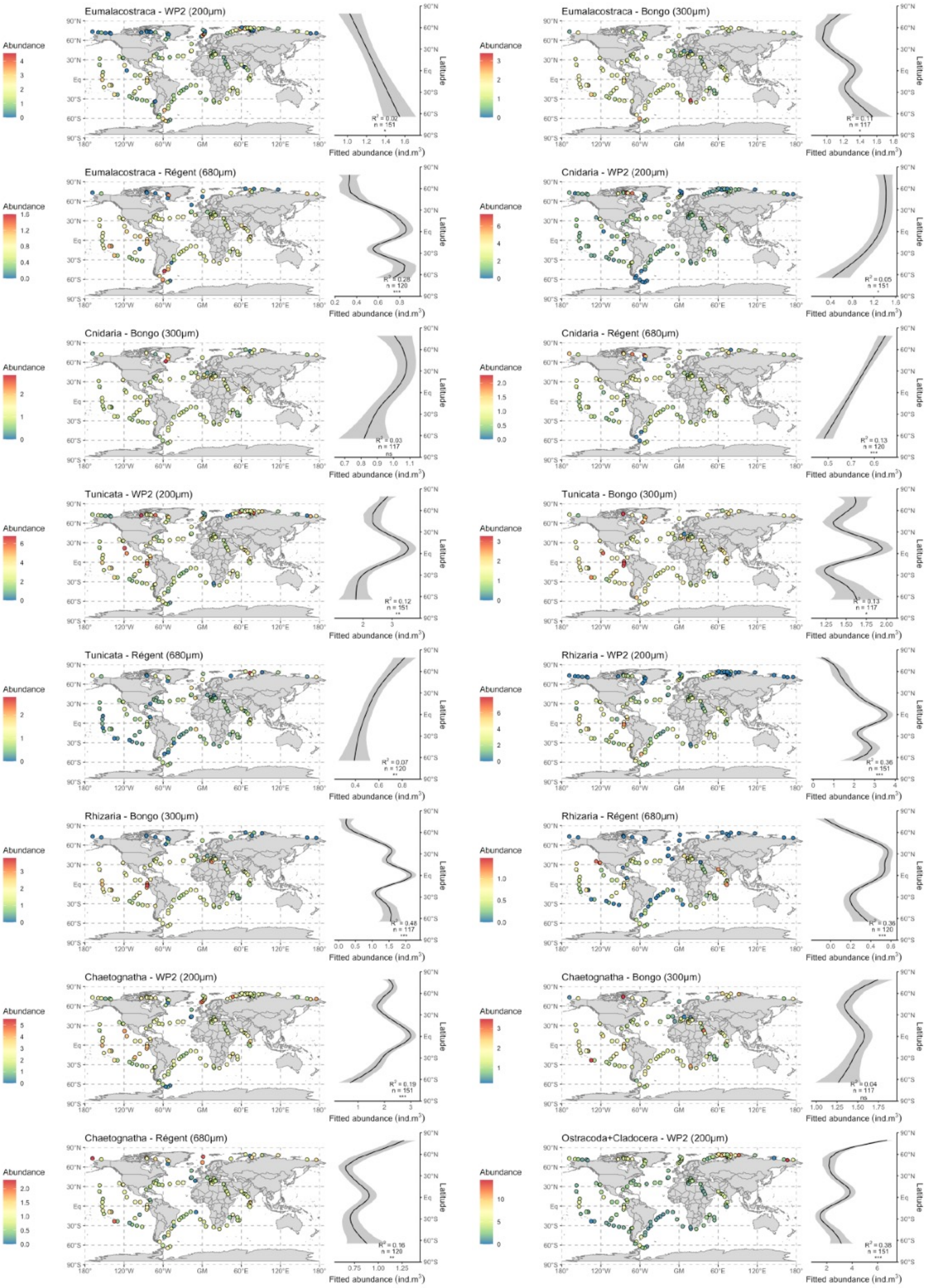

Eumalacostraca - WP2 (200µm)
R^2 = 0.02
n = 151
*
Eumalacostraca - Bongo (300µm)
R^2 = 0.11
n = 117
*
Eumalacostraca - Régent (680µm)
R^2 = 0.28
n = 120
***
Cnidaria - WP2 (200µm)
R^2 = 0.05
n = 151
*
Cnidaria - Bongo (300µm)
R^2 = 0.03
n = 117
ns
Cnidaria - Régent (680µm)
R^2 = 0.13
n = 120
***
Tunicata - WP2 (200µm)
R^2 = 0.12
n = 151
**
Tunicata - Bongo (300µm)
R^2 = 0.13
n = 117
*
Tunicata - Régent (680µm)
R^2 = 0.07
n = 120
**
Rhizaria - WP2 (200µm)
R^2 = 0.36
n = 151
***
Rhizaria - Bongo (300µm)
R^2 = 0.48
n = 117
***
Rhizaria - Régent (680µm)
R^2 = 0.36
n = 120
***
Chaetognatha - WP2 (200µm)
R^2 = 0.19
n = 151
***
Chaetognatha - Bongo (300µm)
R^2 = 0.04
n = 117
ns
Chaetognatha - Régent (680µm)
R^2 = 0.16
n = 120
**
Ostracoda+Cladocera - WP2 (200µm)
R^2 = 0.38
n = 151
***
Abundance
Latitude
Fitted abundance (ind.m^3)

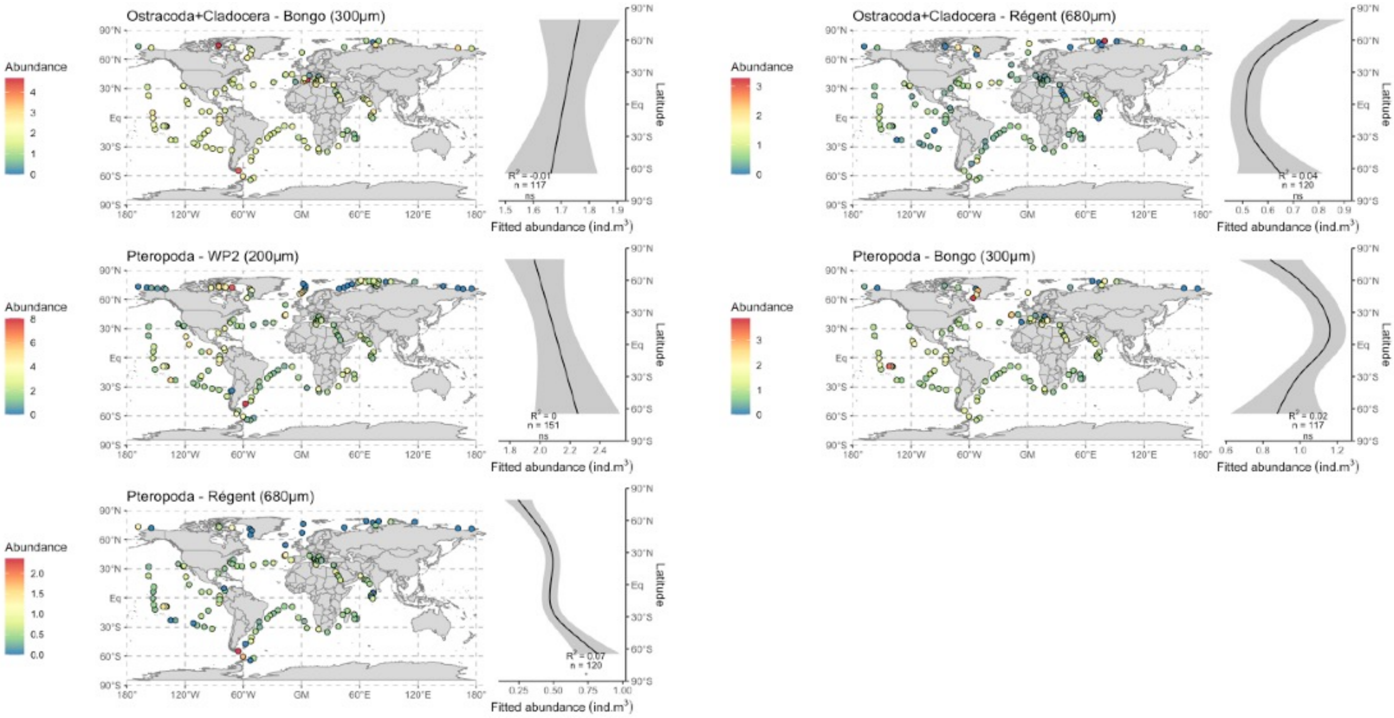

Supplementary Figure S2: Maps and latitudinal patterns of the abundance (cubic-transformed ind.m3) of the zooplankton groups sampled with the three plankton nets (WP2, Bongo and Régent). The solid curves on the zonal side plots illustrate the prediction from the Generalized Additive Model (GAM) fitting transformed abundance against latitude. The explanatory power of the GAM (adjusted R^2), the number of samples used and the significance of the smooth term ($p < 0.001$ = ***, $p < 0.01$ = ***, $p < 0.05$ = *, $p > 0.05$ = ns) are reported on the plots. The grey ribbon illustrates the standard error of the prediction.

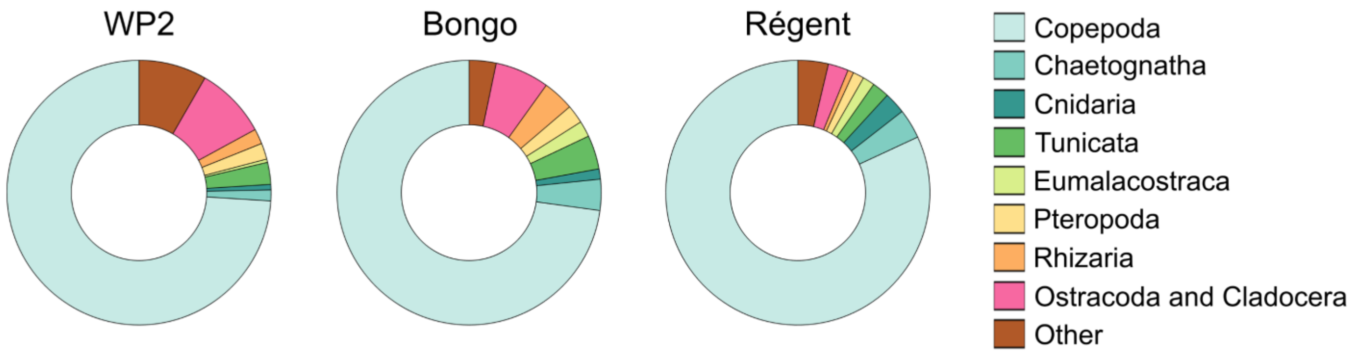

Net	Copepoda	Chaetognatha	Cnidaria	Tunicata	Eumalacostraca	Pteropoda	Rhizaria	Ostracoda + Cladocera	Other
**WP2 (200µm)**	73.99	1.34	0.66	2.69	0.40	1.97	1.85	8.77	8.32
**Bongo (300µm)**	72.85	3.79	1.25	4.14	1.95	2.26	3.84	6.63	3.30
**Régent (680µm)**	81.90	3.62	2.65	2.09	1.46	1.36	0.72	2.47	3.73

Supplementary Document S3: Distribution of the relative contribution (%) of the main mesozooplankton groups to total zooplankton community abundance across the three plankton nets used.

Supplementary Document S4: Comparing mesh size and sampling depth effects on the abundance distributions of the main zooplankton groups.

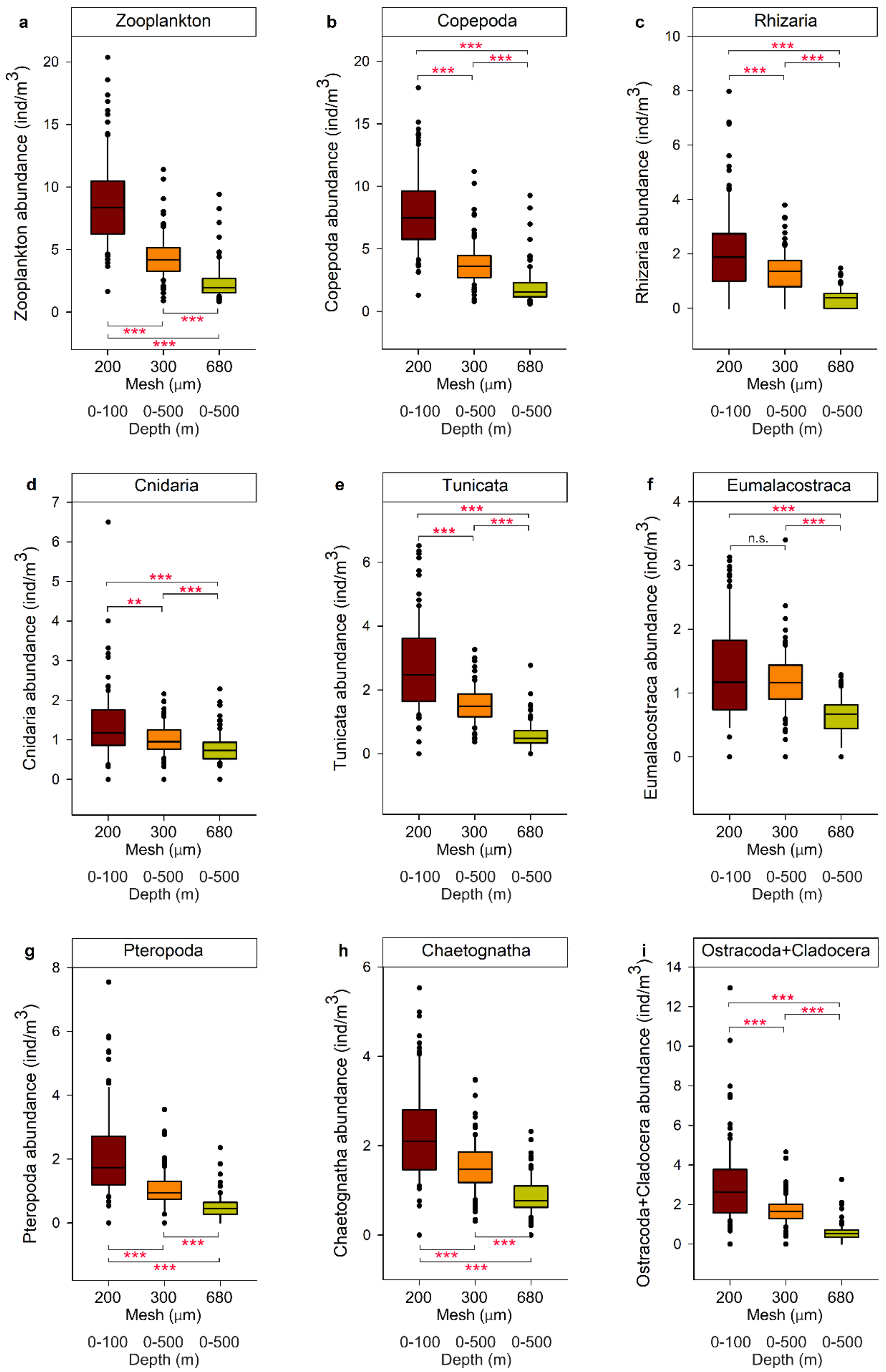

Figure S4.1: Comparing the cubic-transformed abundance distributions of the main zooplankton groups studied between the three plankton mesh sizes (200µm, WP2; 300µm, Bongo; 680µm, Régent) and sampling

depth of the net hauls (0-100m, WP2; 0-500m, Bongo and Régent). a) Total zooplankton, b) Copepoda, c) Rhizaria, d) Cnidaria, e) Tunicata, f) Eumalacostraca, g) Pteropoda, h) Chaetognatha, and i) Ostracoda + Cladocera. For each group and mesh size, non parametric variance analyses (Kruskal-Wallis tests) were performed to test if the nets displayed significant different zooplankton abundances. Conventional labels illustrate the level of significance of the test: n.s. = not significant; ** = p-value < 0.01; *** = p-value < 0.001.

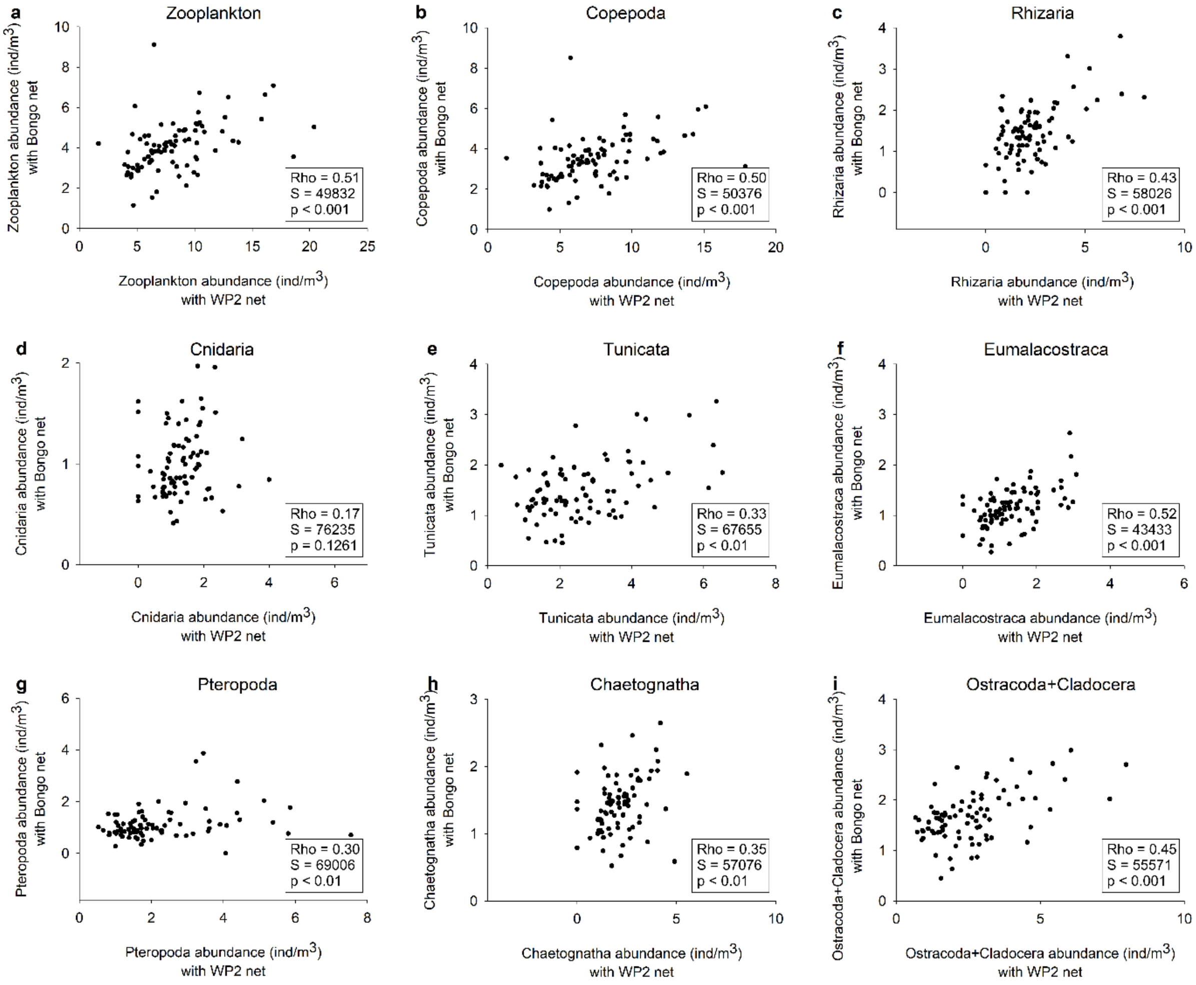

Figure S4.2: Pairwise correlations of cubic-transformed abundance distributions of the main zooplankton groups studied between the sampling depth of the net hauls (0-100m, WP2; 0-500m, Bongo). a) Total zooplankton, b) Copepoda, c) Rhizaria, d) Cnidaria, e) Tunicata, f) Eumalacostraca, g) Pteropoda, h) Chaetognatha, and i) Ostracoda + Cladocera. The results from the Spearman's rank correlation tests are shown on the plots.

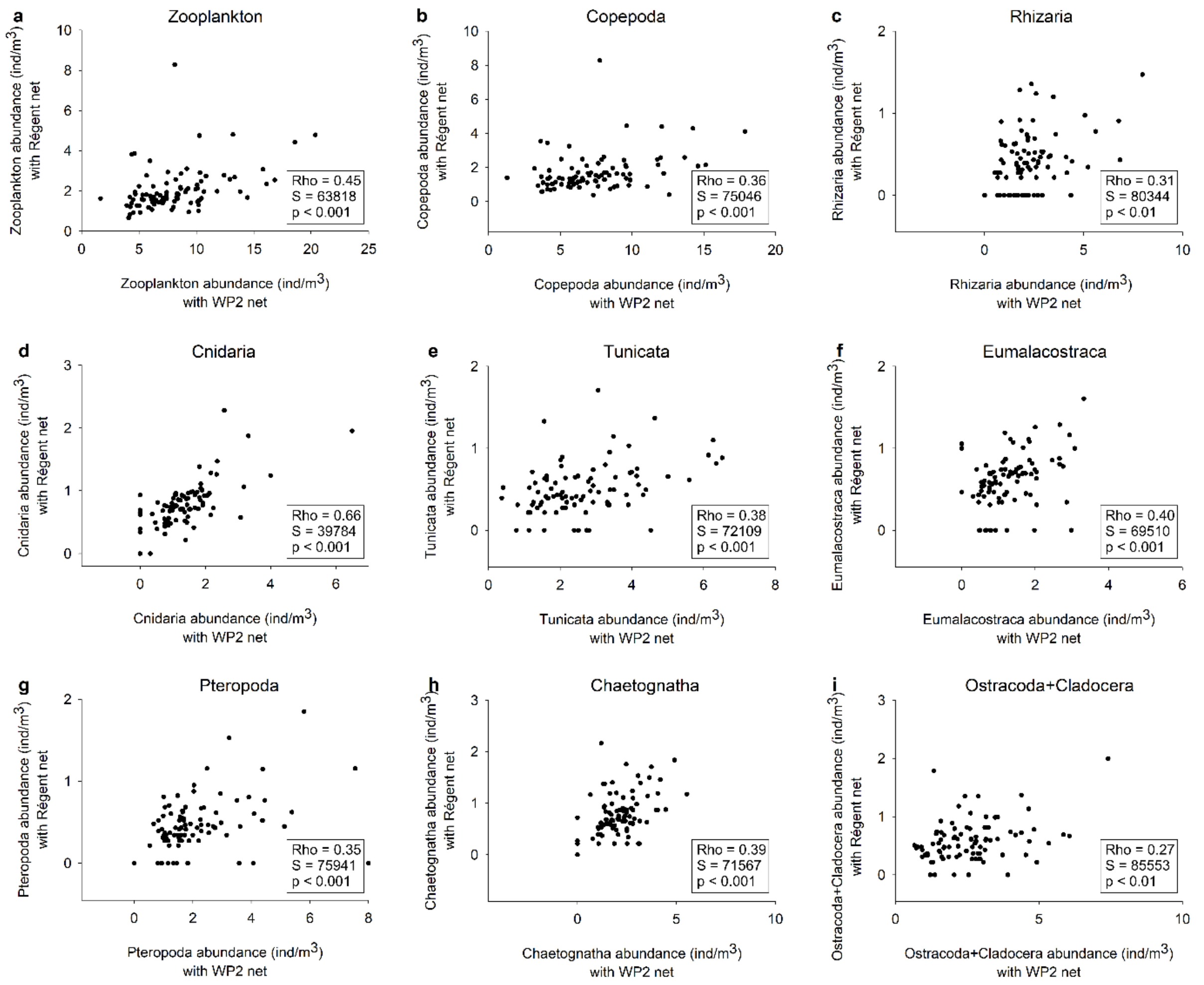

Figure S4.3: Pairwise correlations of cubic-transformed abundance distributions of the main zooplankton groups studied between the sampling depth of the net hauls (0-100m, WP2; 0-500m, Régent). a) Total zooplankton, b) Copepoda, c) Rhizaria, d) Cnidaria, e) Tunicata, f) Eumalacostraca, g) Pteropoda, h) Chaetognatha, and i) Ostracoda + Cladocera. The results from the Spearman's rank correlation tests are shown on the plots.

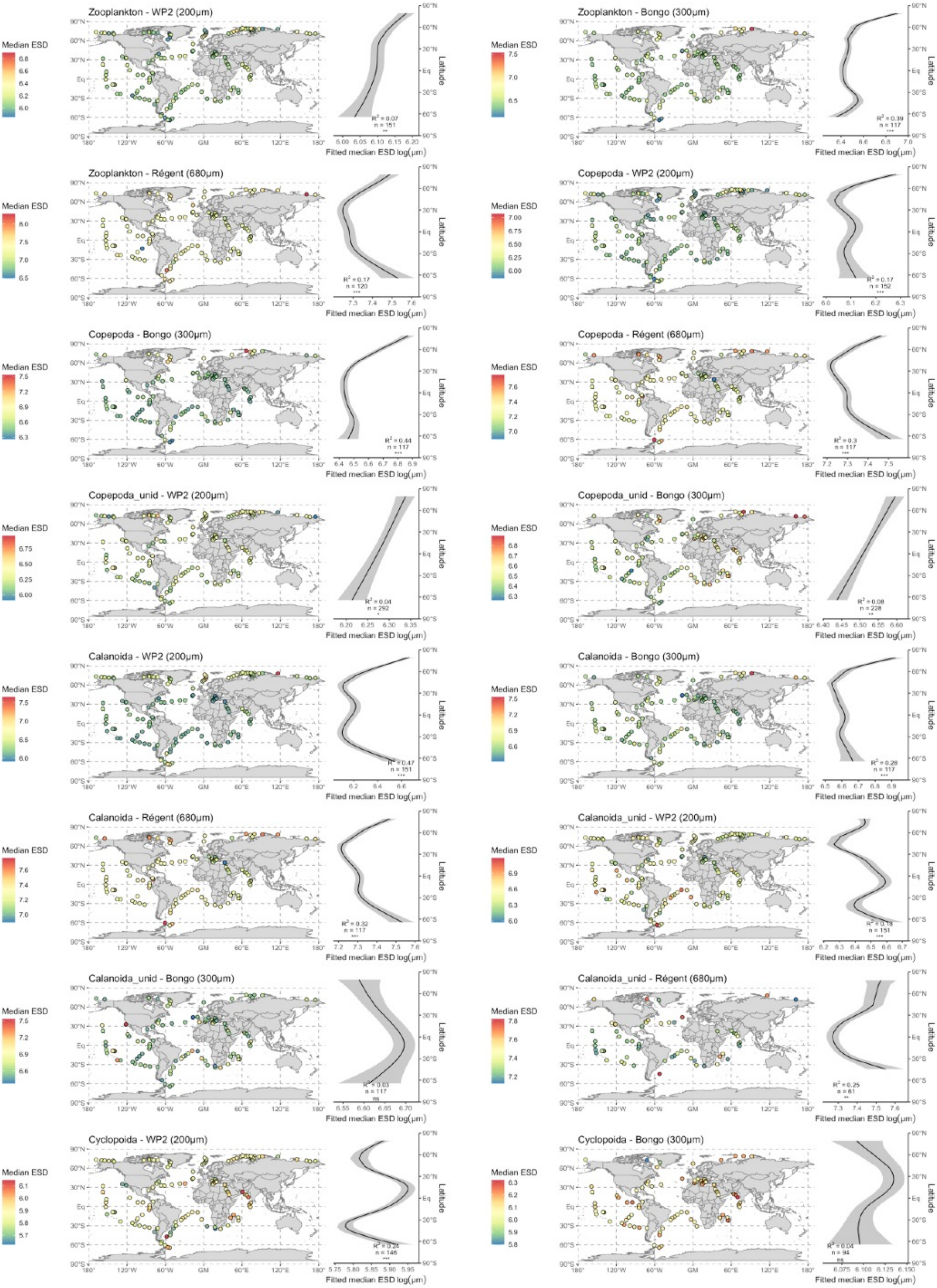

Zooplankton - WP2 (200µm)
Zooplankton - Bongo (300µm)
Zooplankton - Régent (680µm)
Copepoda - WP2 (200µm)
Copepoda - Bongo (300µm)
Copepoda - Régent (680µm)
Copepoda_unid - WP2 (200µm)
Copepoda_unid - Bongo (300µm)
Calanoida - WP2 (200µm)
Calanoida - Bongo (300µm)
Calanoida - Régent (680µm)
Calanoida_unid - WP2 (200µm)
Calanoida_unid - Bongo (300µm)
Calanoida_unid - Régent (680µm)
Cyclopoida - WP2 (200µm)
Cyclopoida - Bongo (300µm)
Median ESD
Latitude
Fitted median ESD log(µm)

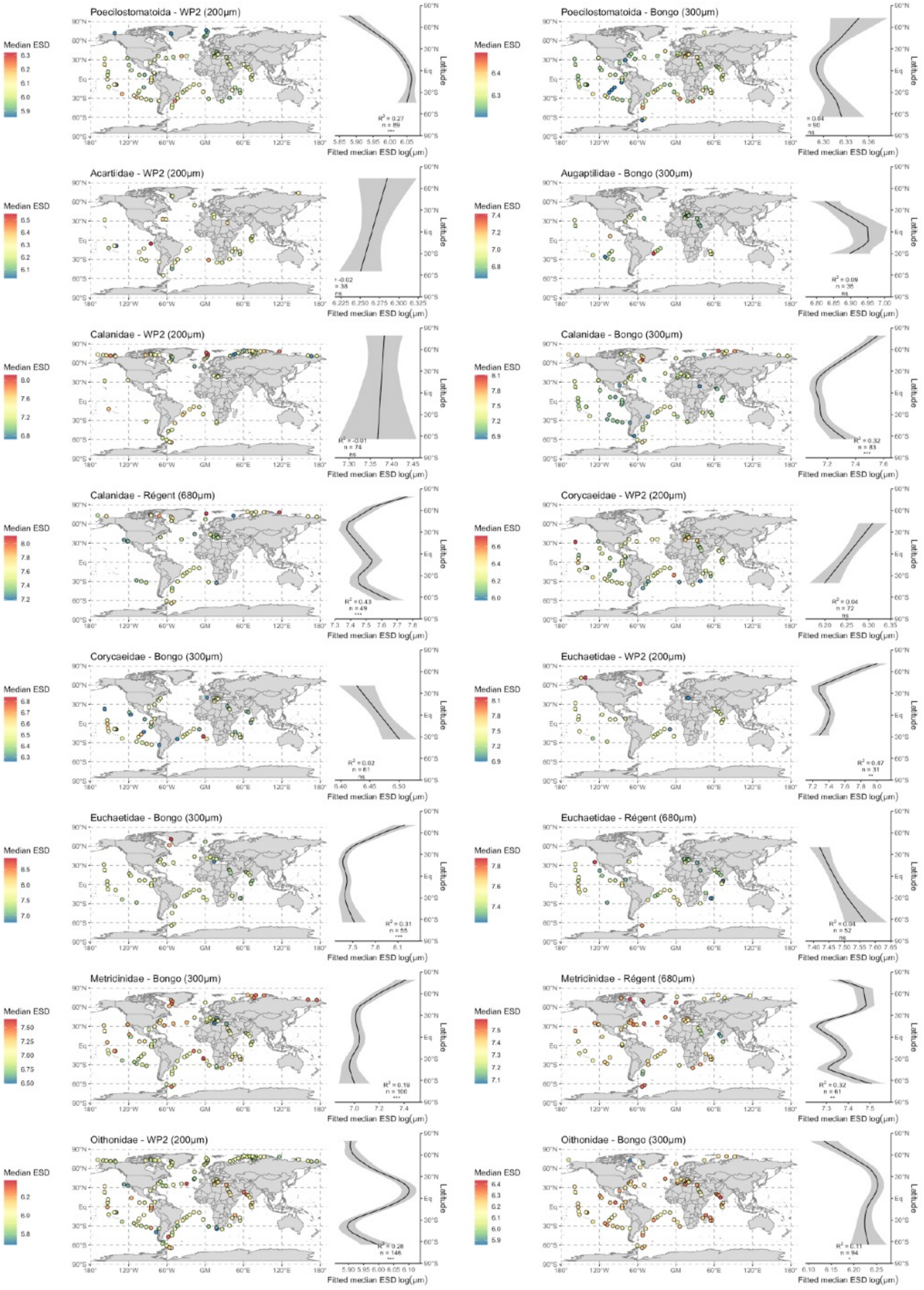

Poecilostomatoida - WP2 (200µm)
Poecilostomatoida - Bongo (300µm)
Acartiidae - WP2 (200µm)
Augaptilidae - Bongo (300µm)
Calanidae - WP2 (200µm)
Calanidae - Bongo (300µm)
Calanidae - Régent (680µm)
Corycaeidae - WP2 (200µm)
Corycaeidae - Bongo (300µm)
Euchaetidae - WP2 (200µm)
Euchaetidae - Bongo (300µm)
Euchaetidae - Régent (680µm)
Metridinidae - Bongo (300µm)
Metridinidae - Régent (680µm)
Oithonidae - WP2 (200µm)
Oithonidae - Bongo (300µm)
Median ESD
Latitude
Fitted median ESD log(µm)
R^2 = 0.27 n = 89
R^2 = 0.09 n = 35 ns
R^2 = -0.01 n = 74 ns
R^2 = 0.32 n = 83
R^2 = 0.43 n = 49
R^2 = 0.04 n = 72 ns
R^2 = 0.02 n = 61 ns
R^2 = 0.47 n = 31
R^2 = 0.31 n = 55
R^2 = 0.04 n = 52 ns
R^2 = 0.19 n = 100
R^2 = 0.32 n = 61
R^2 = 0.28 n = 146
R^2 = 0.11 n = 94

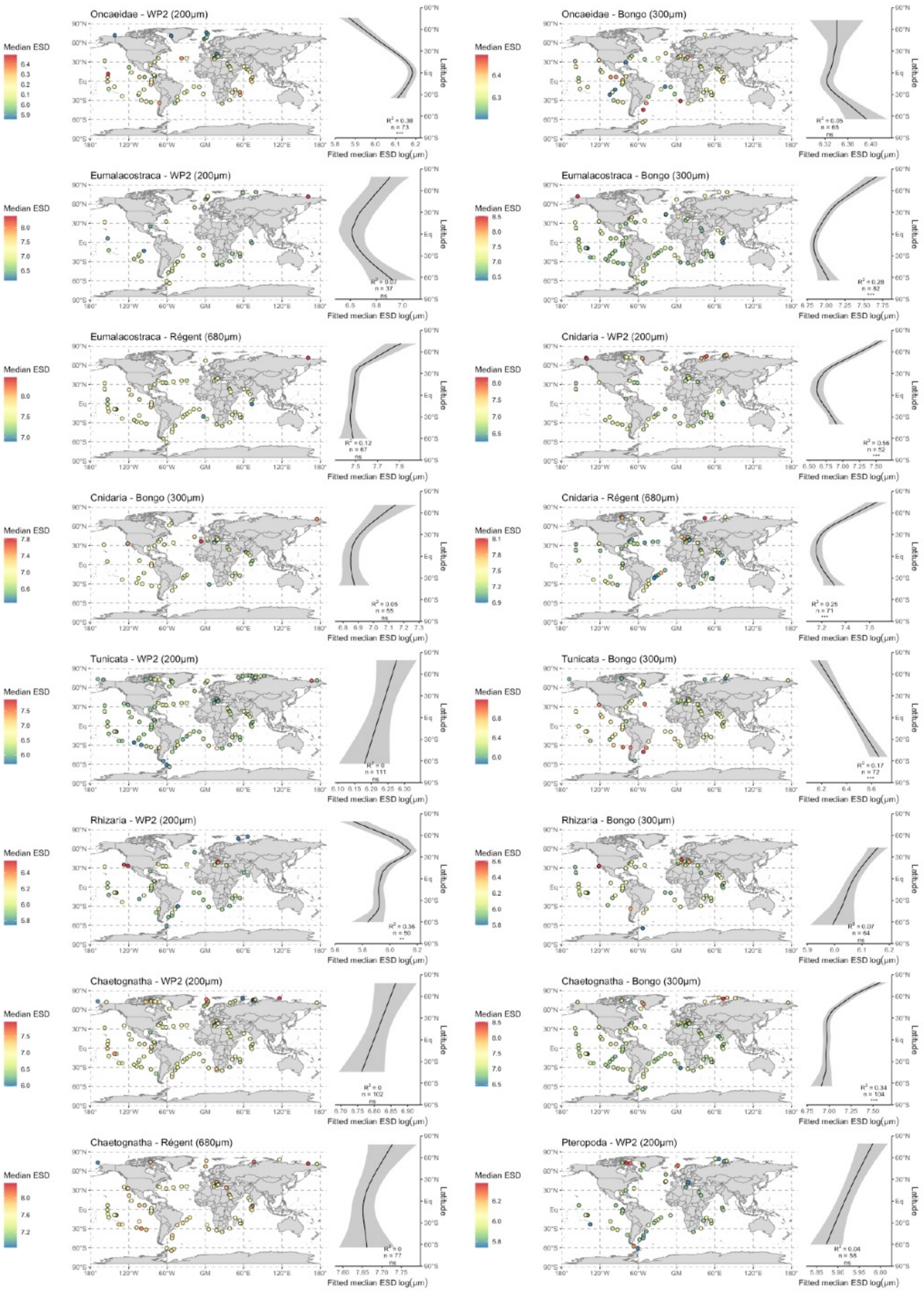
Oncaeidae - WP2 (200µm)
Oncaeidae - Bongo (300µm)
Eumalacostraca - WP2 (200µm)
Eumalacostraca - Bongo (300µm)
Eumalacostraca - Régent (680µm)
Cnidaria - WP2 (200µm)
Cnidaria - Bongo (300µm)
Cnidaria - Régent (680µm)
Tunicata - WP2 (200µm)
Tunicata - Bongo (300µm)
Rhizaria - WP2 (200µm)
Rhizaria - Bongo (300µm)
Chaetognatha - WP2 (200µm)
Chaetognatha - Bongo (300µm)
Chaetognatha - Régent (680µm)
Pteropoda - WP2 (200µm)
Median ESD
Fitted median ESD log(µm)
Latitude

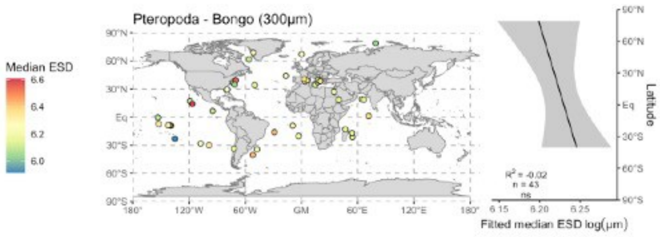

Supplementary Figure S5: Maps and latitudinal patterns of the logged median Equivalent Spherical Diameter (ESD, µm) observed for the various zooplankton groups sampled with the three plankton nets (WP2, Bongo and Régent). The major and minor axes of the best fitting ellipses were measured for each organism to measure their ESD. Community-level size structure was estimated through the median value of the ESD distribution at individual-level. The solid curve on the zonal side plots illustrate the prediction from the Generalized Additive Model (GAM) fitting median ESD as a function of latitude. The explanatory power of the GAM (adjusted R2), the number of samples used and the significance of the smooth term ($p < 0.001$ = ***, $p < 0.01$ = ***, $p < 0.05$ = *, $p > 0.05$ = ns) are reported on the plots. The grey ribbon illustrates the standard error of the prediction. Only the stations where ESD was measured for at least 20 individuals of a group and only the groups with at least 30 stations were modelled here.

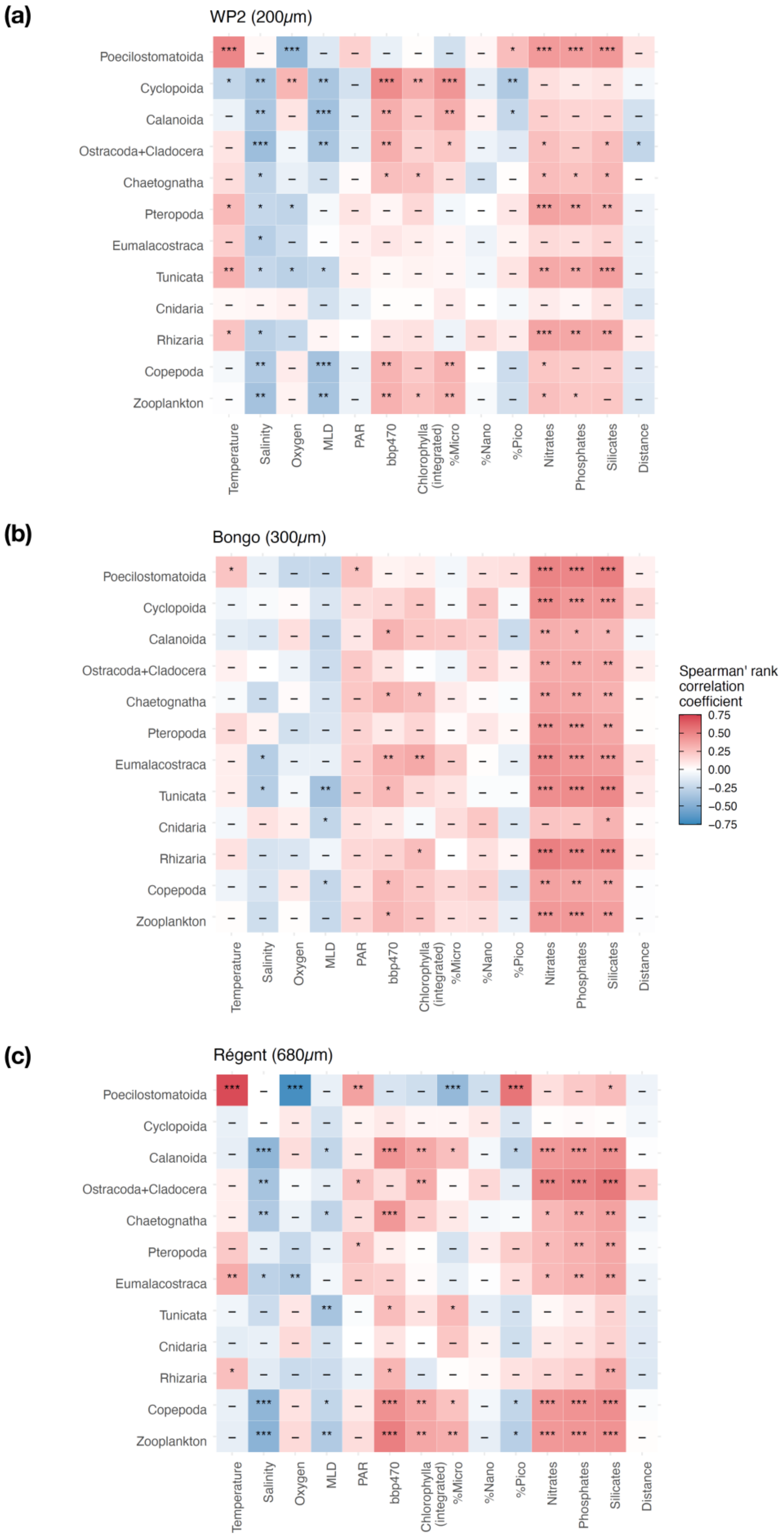
(a)
WP2 (200μm)
Poecilostomatoida
Cyclopoida
Calanoida
Ostracoda+Cladocera
Chaetognatha
Pteropoda
Eumalacostraca
Tunicata
Cnidaria
Rhizaria
Copepoda
Zooplankton
Temperature
Salinity
Oxygen
MLD
PAR
bbp470
Chlorophylla (integrated)
%Micro
%Nano
%Pico
Nitrates
Phosphates
Silicates
Distance
(b)
Bongo (300μm)
(c)
Régent (680μm)
Spearman' rank correlation coefficient
0.75
0.50
0.25
0.00
−0.25
−0.50
−0.75

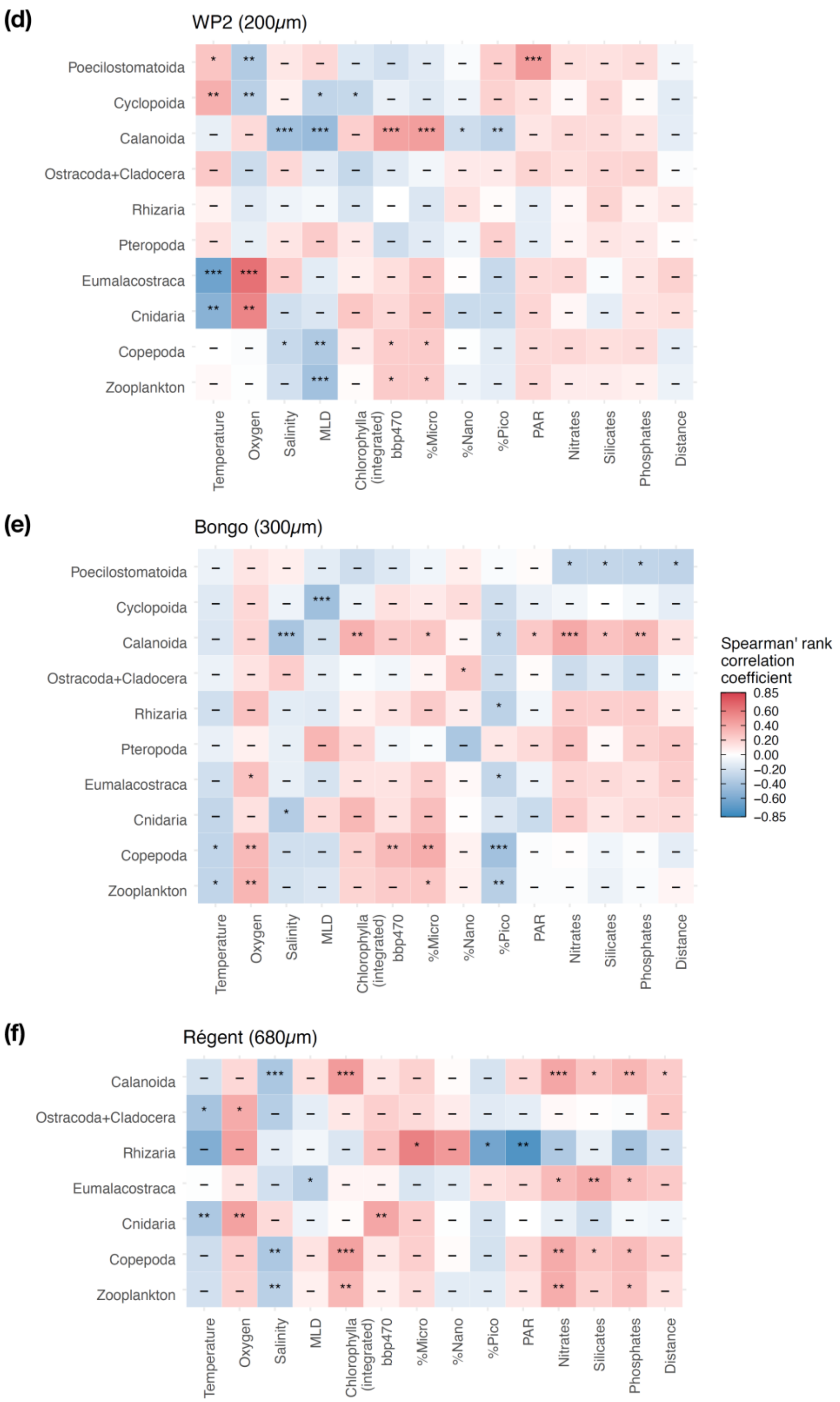

Supplementary Figure S8: Heatmaps of the Spearman's rank correlation coefficients computed between the (a)-(b)-(c) abundance (cubic-transfromed) and the (d)-(e)-(f) median ESD (logged) of the zooplankton groups and the 13 environmental covariates as measured by the (a)-(d) WP2 net (200µm mesh), (b)-(e) Bongo net (300µm mesh) and (c)-(f) Régent net (680µm). The significance of the Spearman's rank correlation tests are reported in the tiles ($p < 0.001$ = ***, $p < 0.01$ = ***, $p < 0.05$ = *, $p > 0.05$ = ns). Only the stations where ESD was measured for at least 20 individuals of a group were considered when computing the correlation coefficients.

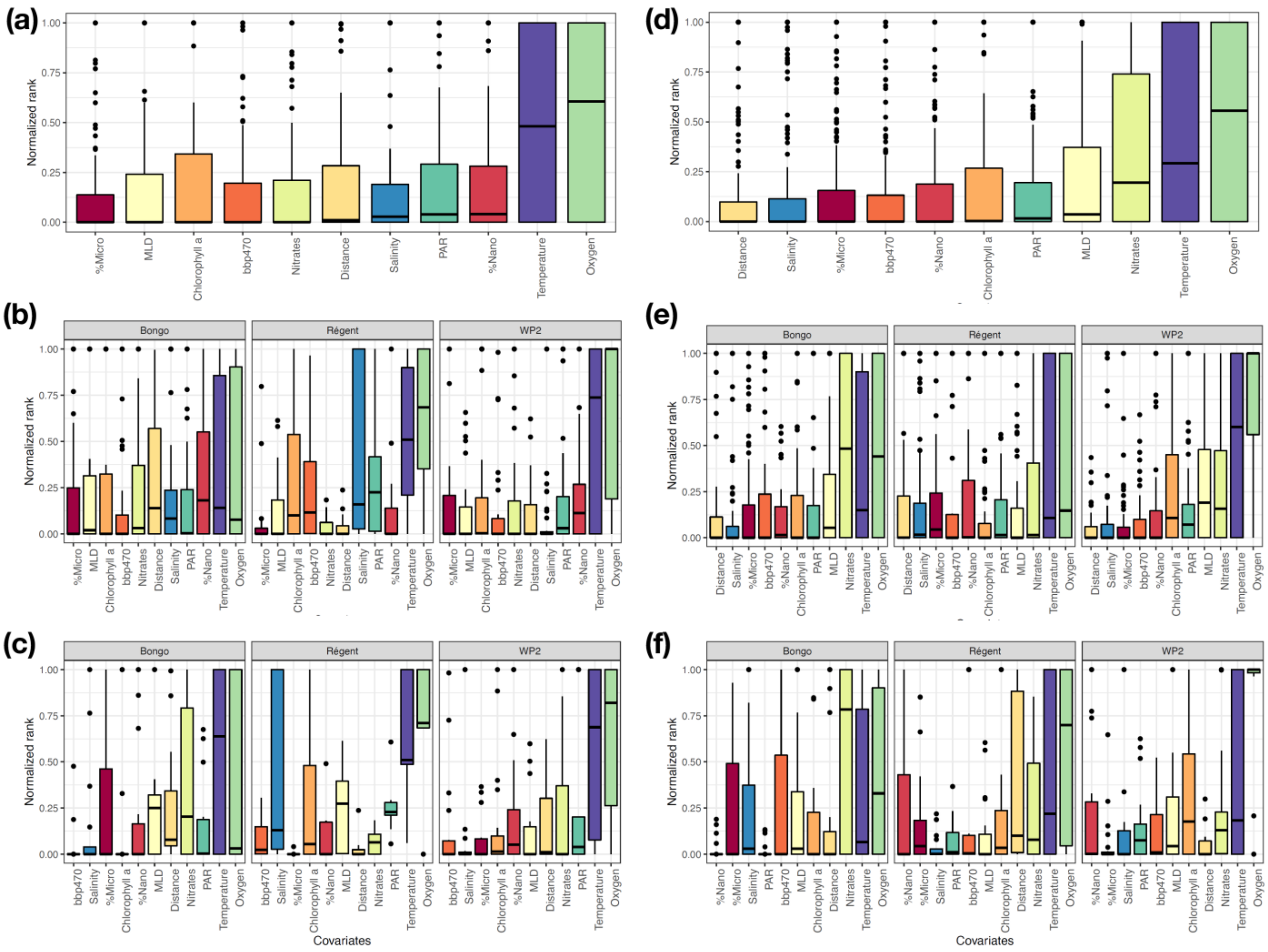

Supplementary Figure S9: Distribution of the normalized ranks of variable significance for the environmental covariates used in the the Generalized Additive Models (GAMs) fit to model the observed (a)-(b)-(c) median ESD (logged) and (d)-(e)-(f) abundance (cubic-transformed) measured for all zooplankton groups based on: (a)-(d) all plankton nets together (covariates are displayed in order of increasing significance of median normalized rank); (b)-(e) per net type; and (c)-(f) based on the groups shown in Tables 1 and 2. Ranks range between 0 (least important covariate) and 1 (most important covariate). The F statistic of the smooth term associated to each covariate was extracted and was used to quantitatively rank the covariates according to their relative significance (the higher the F value, the more significant) within their corresponding GAM. For each GAM, ranks were normalized by dividing the F value of every smooth term by the maximum F value across al smooth terms. The complete statistics of the GAMs are given in Supplementary Table S7.

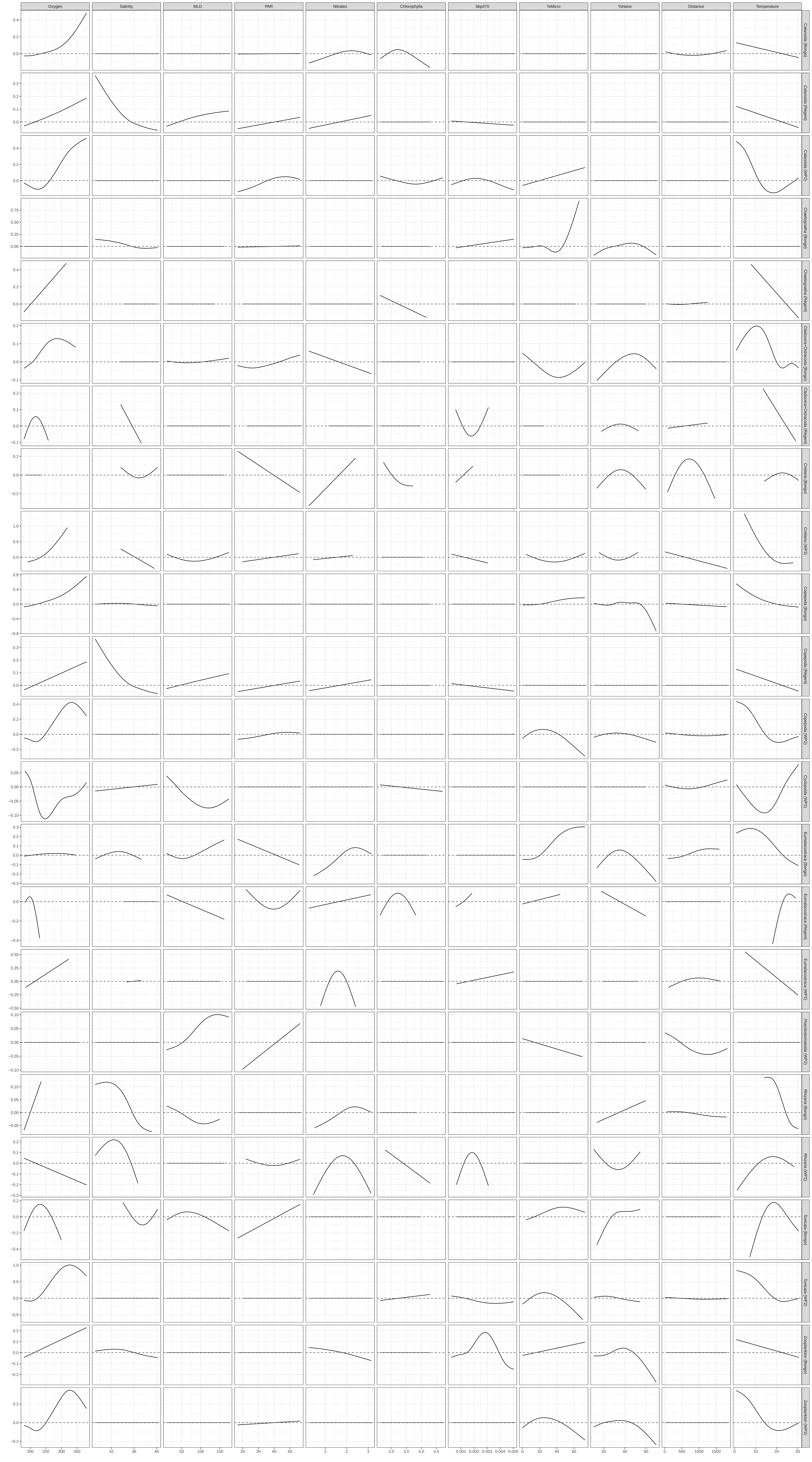

Oxygen
Salinity
MLD
PAR
Nitrates
Chlorophylla
bbp470
%Micro
%Nano
Distance
Temperature
Calanoida (Bongo)
Calanoida (Régent)
Calanoida (WP2)
Chaetognatha (Bongo)
Chaetognatha (Régent)
Cladocera+Ostracoda (Bongo)
Cladocera+Ostracoda (Régent)
Cnidaria (Bongo)
Cnidaria (WP2)
Copepoda (Bongo)
Copepoda (Régent)
Copepoda (WP2)
Cyclopoida (WP2)
Eumalacostraca (Bongo)
Eumalacostraca (Régent)
Eumalacostraca (WP2)
Poecilostomatoida (WP2)
Rhizaria (Bongo)
Rhizaria (WP2)
Tunicata (Bongo)
Tunicata (WP2)
Zooplankton (Bongo)
Zooplankton (WP2)

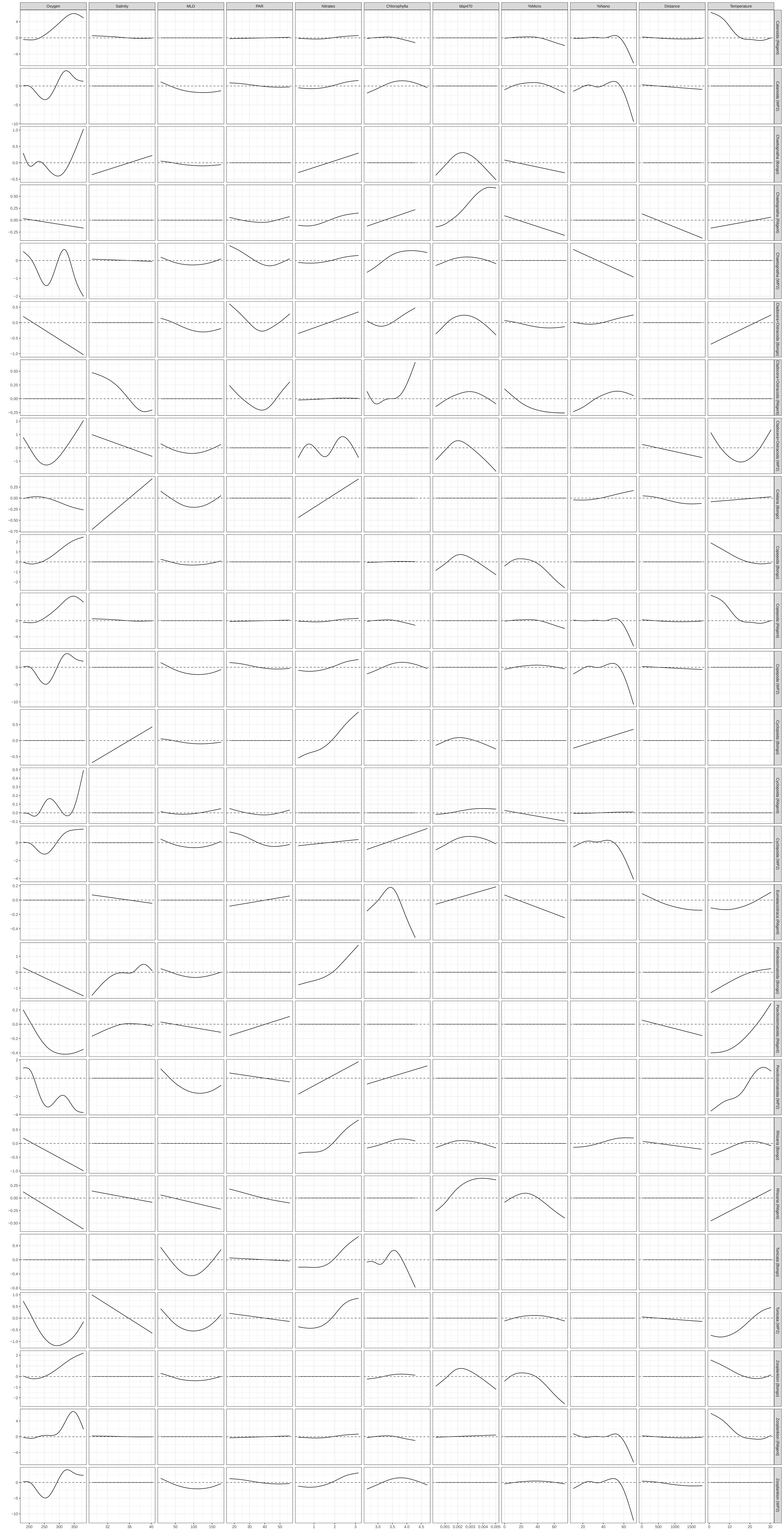

Oxygen
Salinity
MLD
PAR
Nitrates
Chlorophylla
bbp470
%Micro
%Nano
Distance
Temperature
Calanoida (Régent)
Calanoida (WP2)
Chaetognatha (Bongo)
Chaetognatha (Régent)
Chaetognatha (WP2)
Cladocera+Ostracoda (Bongo)
Cladocera+Ostracoda (Régent)
Cladocera+Ostracoda (WP2)
Cnidaria (Bongo)
Copepoda (Bongo)
Copepoda (Régent)
Copepoda (WP2)
Cyclopoida (Bongo)
Cyclopoida (Régent)
Cyclopoida (WP2)
Eumalacostraca (Régent)
Poecilostomatoida (Bongo)
Poecilostomatoida (Régent)
Poecilostomatoida (WP2)
Rhizaria (Bongo)
Rhizaria (Régent)
Tunicata (Bongo)
Tunicata (WP2)
Zooplankton (Bongo)
Zooplankton (Régent)
Zooplankton (WP2)

Supplementary Document S12: Comparing zooplankton groups abundance estimates between stations located within Eastern Boundary Upwelling Systems (EBUS) and outside EBUS.

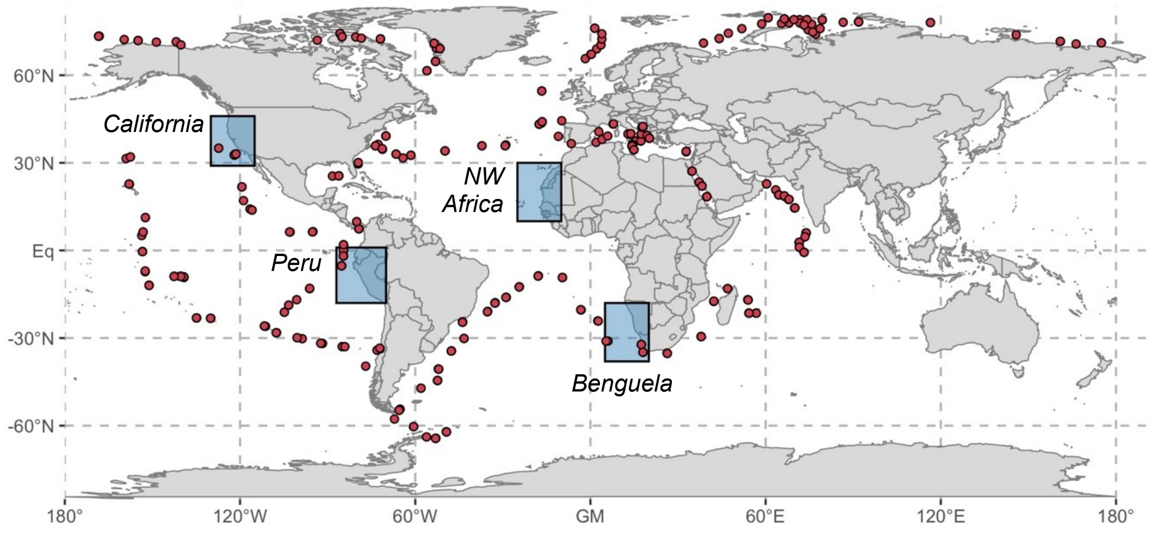

Figure S12.1: Geographic position of the Tara Oceans sampling stations (in red) from which the zooplankton abundance estimates used in our study come from and the position of the major Eastern Boundary Upwelling Systems (EBUS). The extent for the spatial boundary boxes are based on those shown in Figure 2 of Chavez & Messié (2009). Any sampling station whose spatial coordinates fell into one of the four EBUS was labelled as a "EBUS station". The corresponding abundance estimates of the main zooplankton groups were compared to those stemming from the "non EBUS stations" that displayed a similar latitudinal range (i.e. 29°N-46°N and -30°N-1°N) as the EBUS stations (see below). This way, high latitude sampling stations were discarded from this analysis so we can focus on those abundance patterns that mostly reflect effects due to the EBUS conditions.

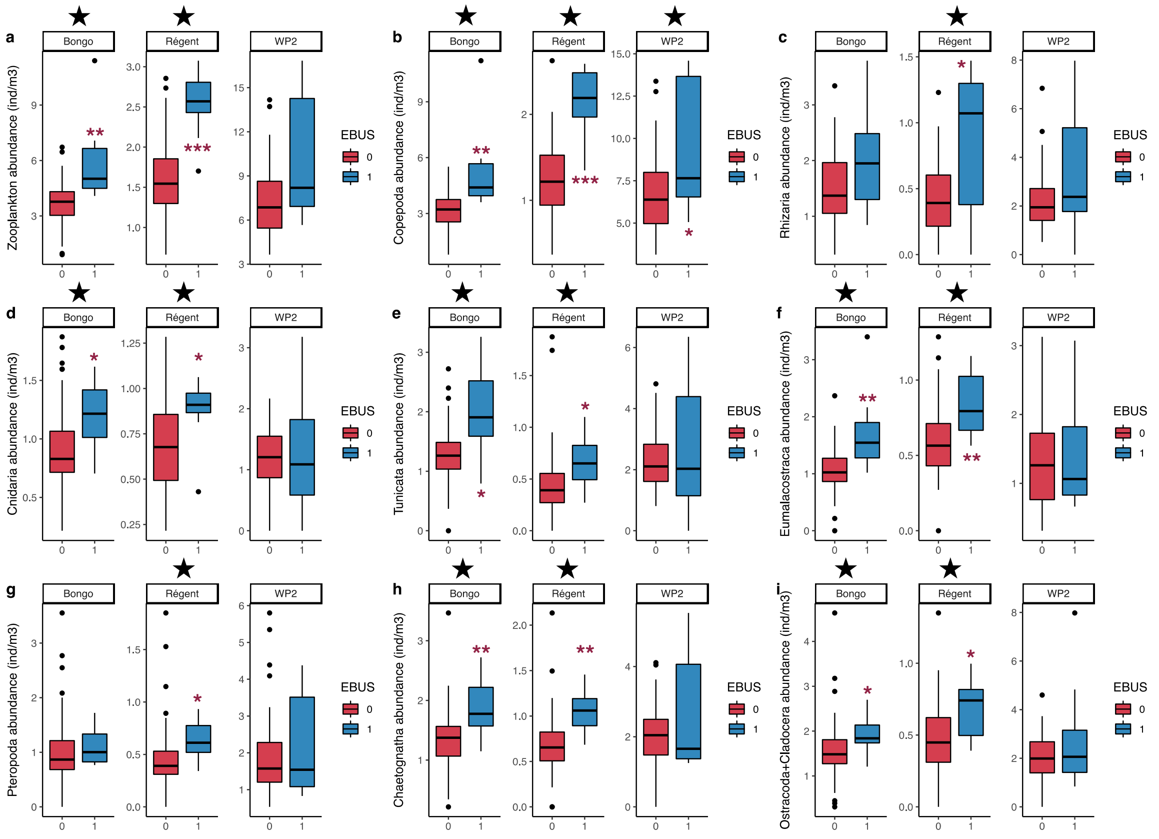

Figure S12.2: Comparing the cubic-transformed abundance distributions of the main zooplankton groups studied across three plankton nets (WP2, 200µm; Bongo, 300µm; Régent, 680µm) between the EBUS stations in blue (see Fig. SXX1) and the comparable non EBUS stations in red (i.e. stations located between 29°N-46°N and -30°N-1°N). a) Total zooplankton, b) Copepoda, c) Rhizaria, d) Cnidaria, e) Tunicata, f) Eumalacostraca, g) Pteropoda, h) Chaetognatha, and i) Ostracod + Cladocera. For each group and net type, non parametric variance analyses (Kruskal-Wallis tests) were performed to test if the EBUS stations displayed significant higher zooplankton abundances. The groups and nets for which a significant (p-value < 0.05) was found between EBUS and non EBUS stations are highlighted with a black star. Conventional labels illustrate the level of significance of the test: * = p-value < 0.05; ** = p-value < 0.01; *** = p-value < 0.001.

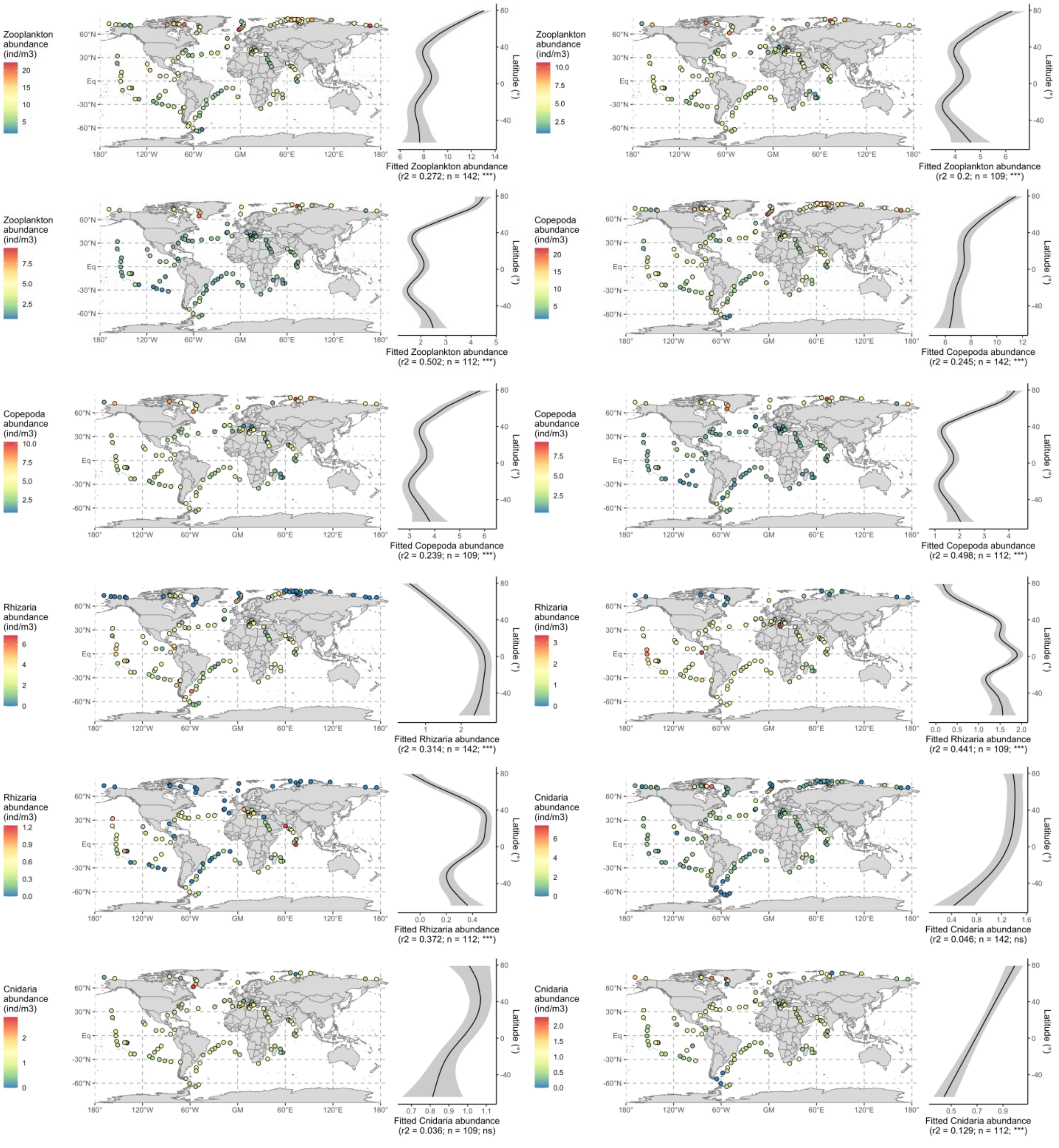

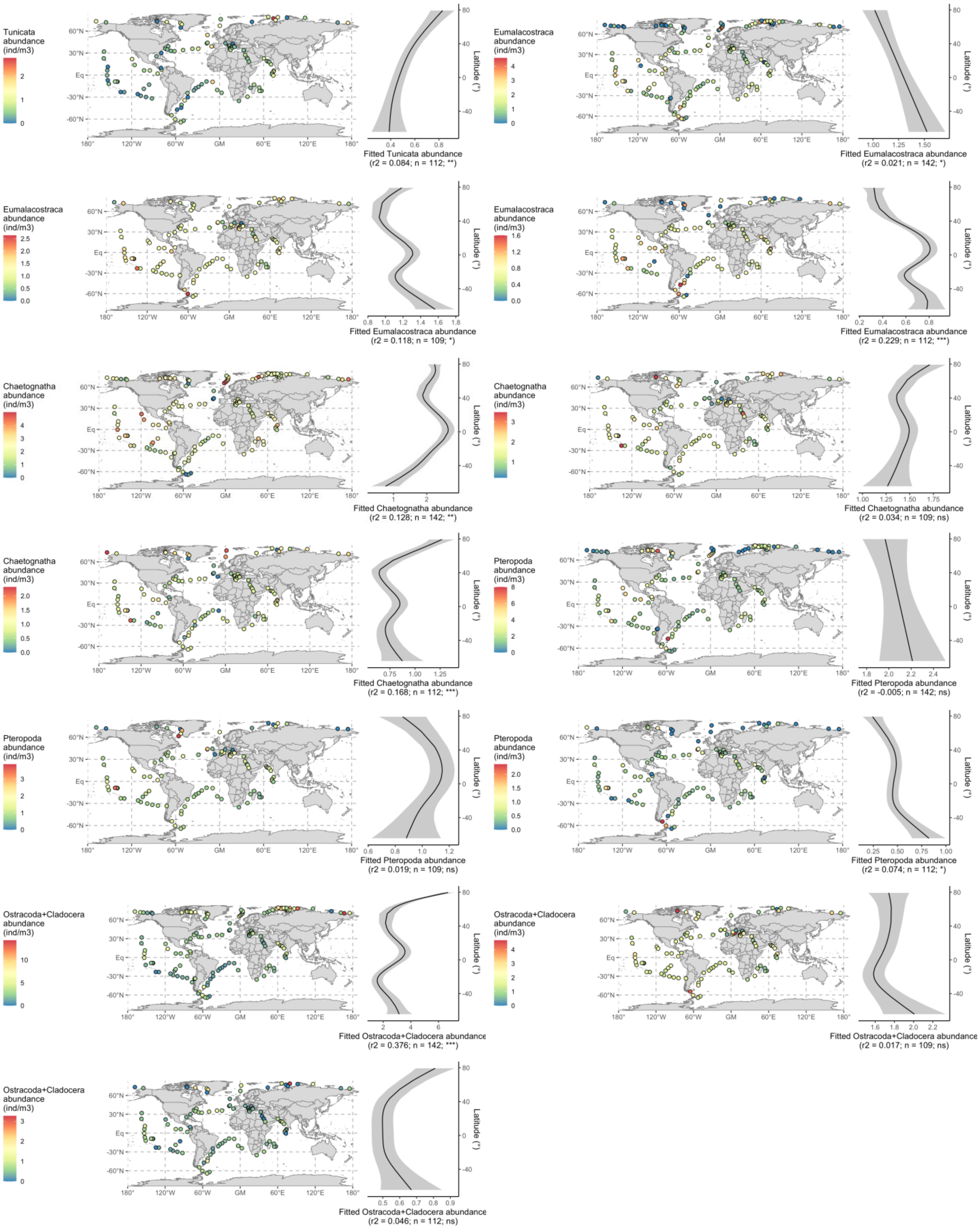

Tunicata abundance (ind/m3)
Fitted Tunicata abundance
(r2 = 0.084; n = 112; **)
Eumalacostraca abundance (ind/m3)
Fitted Eumalacostraca abundance
(r2 = 0.021; n = 142; *)
Eumalacostraca abundance (ind/m3)
Fitted Eumalacostraca abundance
(r2 = 0.118; n = 109; *)
Eumalacostraca abundance (ind/m3)
Fitted Eumalacostraca abundance
(r2 = 0.229; n = 112; ***)
Chaetognatha abundance (ind/m3)
Fitted Chaetognatha abundance
(r2 = 0.128; n = 142; **)
Chaetognatha abundance (ind/m3)
Fitted Chaetognatha abundance
(r2 = 0.034; n = 109; ns)
Chaetognatha abundance (ind/m3)
Fitted Chaetognatha abundance
(r2 = 0.168; n = 112; ***)
Pteropoda abundance (ind/m3)
Fitted Pteropoda abundance
(r2 = -0.005; n = 142; ns)
Pteropoda abundance (ind/m3)
Fitted Pteropoda abundance
(r2 = 0.019; n = 109; ns)
Pteropoda abundance (ind/m3)
Fitted Pteropoda abundance
(r2 = 0.074; n = 112; *)
Ostracoda+Cladocera abundance (ind/m3)
Fitted Ostracoda+Cladocera abundance
(r2 = 0.376; n = 142; ***)
Ostracoda+Cladocera abundance (ind/m3)
Fitted Ostracoda+Cladocera abundance
(r2 = 0.017; n = 109; ns)
Ostracoda+Cladocera abundance (ind/m3)
Fitted Ostracoda+Cladocera abundance
(r2 = 0.046; n = 112; ns)
Latitude (°)
180° 120°W 60°W GM 60°E 120°E 180°
60°N 30°N Eq -30°N -60°N

Figure S12.3: Maps and latitudinal patterns of the abundance (cubic-transformed ind.m3) of the main zooplankton groups sampled with three plankton nets (WP2, 200µm; Bongo, 300µm; Régent, 680µm), excluding those sampling stations that fall within an eastern boundary upwelling system (EBUS). The solid curves on the zonal side plots illustrate the prediction from the Generalized Additive Model (GAM) fitting transformed abundance against latitude. The explanatory power of the GAM (adjusted R2), the number of samples used and the significance of the smooth term ($p < 0.001$ = ***, $p < 0.01$ = ***, $p < 0.05$ = *, $p > 0.05$ = ns) are reported on the plots. The grey ribbon illustrates the standard error of the prediction. As in Supplementary Figure S2, the order of appearance of the groups' maps+plots panel is always as follows: WP2 data, Bongo data and then Régent data.

The effect of the EBUS stations on the cubic-transformed abundance latitudinal patterns modelled above can be examined by comparing these maps and zonal latitudinal plots to their counterparts shown in Supplementary Figure S2. Very few differences were found, which suggests that EBUS have a relative weak effect on the macroscale patterns of zooplankton abundances. However, we acknowledge that the sampling design of the *Tara* Oceans cruises does not allow to fully investigate this effect, as no stations sampled the northwestern African EBUS and relatively few stations were taken in the other three EBUS (Fig. S12.1). The most notable differences in latitudinal abundance patterns were found for the:

- **Copepoda (WP2)**: the modelled increase in abundances near the equator was way less marked as the relatively high abundances observed in the Peru and Benguela EBUS were discarded.
- **Rhizaria (WP2)**: the modelled decrease in abundances near 30°S was removed as the relatively low abundances observed in the Benguela EBUS were discarded.
- **Tunicata (WP2)**: the modelled increase in abundances near the equator was less marked as the relatively high abundances observed in the Peru EBUS were discarded.

All the other abundance latitudinal patterns modelled were left unchanged by the removal of the EBUS stations. Therefore, although the EBUS seem to have a positive influence of the abundances of most of the main zooplankton groups studied here (Fig. S12.2), they do not strongly impact the modelled latitudinal gradients in zooplankton abundance.

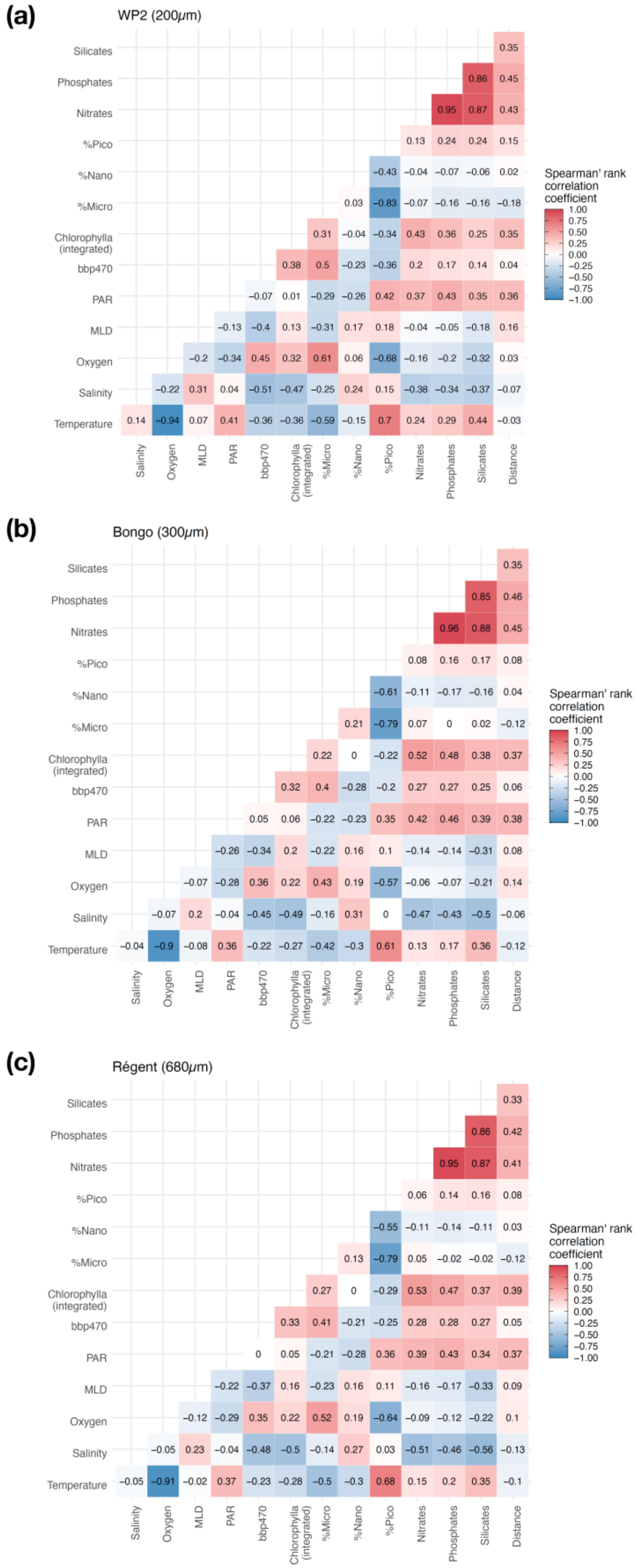

Supplementary Figure S14: Heatmaps of the Spearman’s rank correlation coefficients computed between the 13 covariates as measured at the stations sampled with the (a) WP2 net, (b) Bongo net, and (c) Régent net. Macronutrients (Nitrates, Phosphates and Silicates) concentrations were cubic-transformed and integrated Chlorophyll a concentration was log-transformed. See Methods for details regarding to the in situ measurements of these covariates.

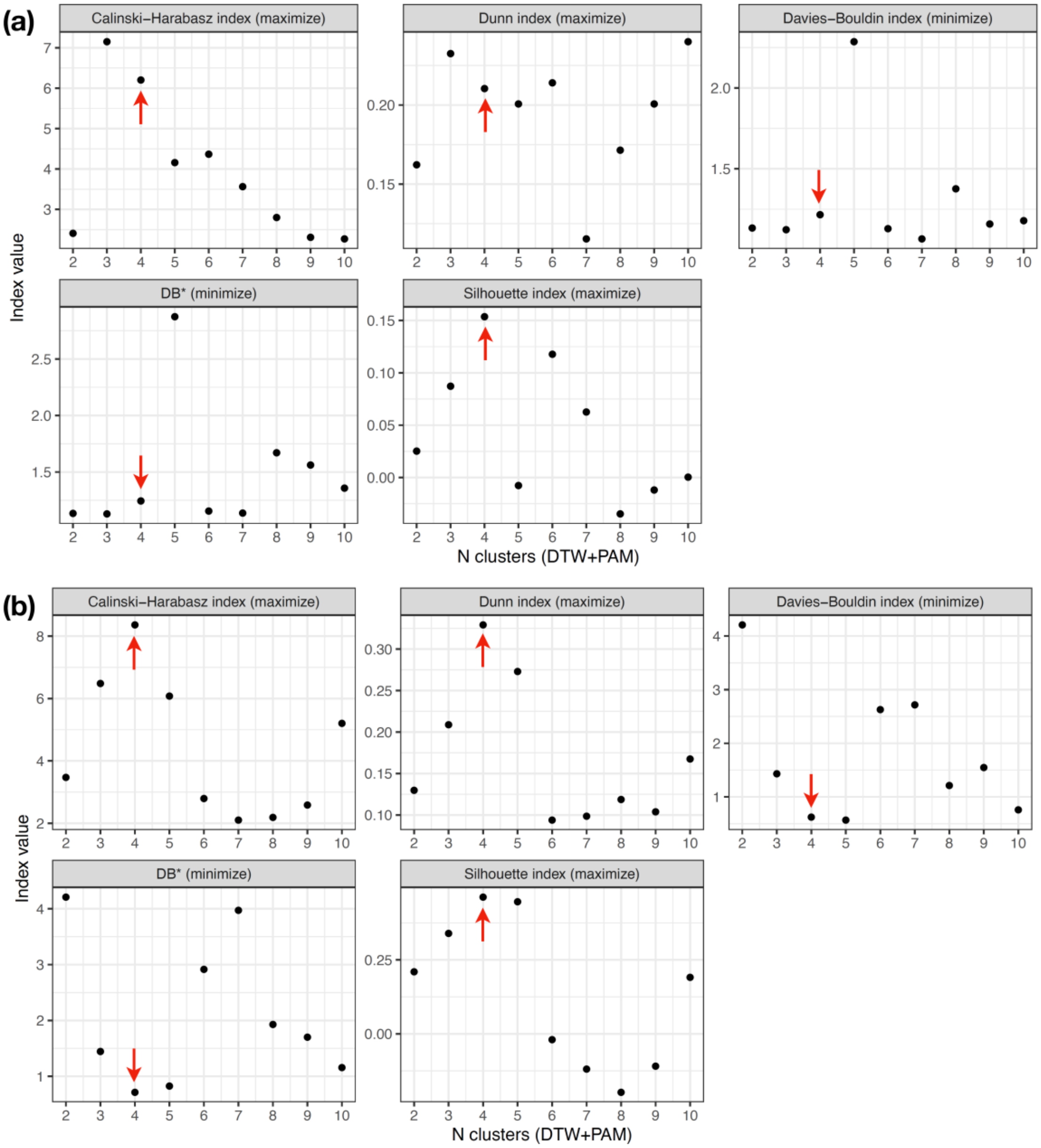

Supplementary Figure S15: Profiles of the five indices of clustering quality measured for 2 to 10 clusters obtained by computing the *Dynamic Time Warping* (DTW) distance and performing Partitioning Around Medoids (PAM) clustering on the smooth curves resulting from the Generalized Additive Models (GAMs) fit on (a) the median ESD estimates of the zooplankton groups highlighted in Table 1, and (b) the abundance of the groups highlighted in Table 2 (one smooth curve per environmental covariate included in the GAMs; clustering was performed on a the multivariate data series obtained by stacking the smooth curves; only GAMs displaying a deviance explained >40% were considered). The red arrows highlight our final choice of the number of cluster which stems from the trade-off across the five indices examined. DB* corresponds to the modified version of the Davies-Bouldin index. Indices values were obtained through the *cvi* function of the *dtwclust* R package. Whether maximum or minimum index values indicate higher or lower cluster quality is specified.
