# Supplementary material for: Macroscale patterns of oceanic zooplankton composition and size structure
Source: Sci Rep. 2021 Aug 3;11:15714. doi: 10.1038/s41598-021-94615-5 (PMC8333327; doi:10.1038/s41598-021-94615-5)
Supplement: Supplementary file 5 — Supplementary Information 5. [file 41598_2021_94615_MOESM5_ESM.docx]

**Supplementary Table S6**: Summary of the Spearman’s rank correlations computed for the zooplankton groups displaying significant correlations between their cubic-transformed abundance (ind.m^3^) and their log-transformed median Equivalent Spherical Diameter (ESD, µm).

**Supplementary Table S7**: Summarizing statistics of the Generalized Additive Models (GAMs) fitted to the global patterns of cubic-transformed abundance (ind.m^3^) and log-transformed median Equivalent Spherical Diameter (ESD, µm) of all zooplankton groups.

**Supplementary Table S13**: Classification of the labels used in the ZooScan imaging system to identify the living zooplankton individuals and the corresponding final zooplankton group(s) used in the present study.
